# Synthesis and Characterization of Supported Mixed MoW Carbide Catalysts

**DOI:** 10.1021/acs.jpcc.2c08352

**Published:** 2023-04-17

**Authors:** M. Führer, T. van Haasterecht, E. J. J. de Boed, P. E. de Jongh, J. H. Bitter

**Affiliations:** † Biobased Chemistry and Technology, Wageningen University, P.O. Box 17, 6700 AA Wageningen, The Netherlands; ‡ Materials Chemistry and Catalysis, Debye Institute for Nanomaterials Science, Utrecht University, Universiteitsweg 99, 3584 CG Utrecht, The Netherlands

## Abstract

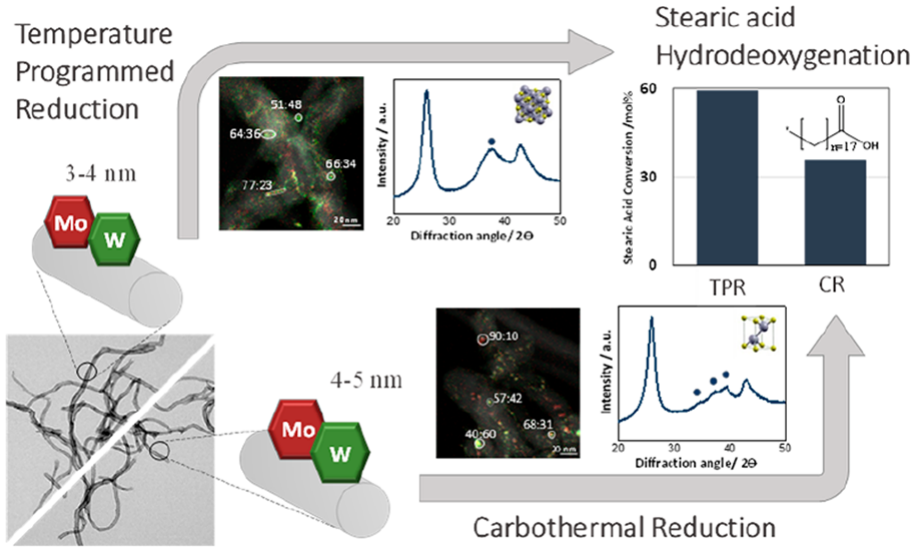

For mixed MoW carbide catalysts, the relationship between synthesis conditions, evolution of (mixed) phases, extent of mixing, and catalytic performance of supported Mo/W carbides remains unclear. In this study, we prepared a series of carbon nanofiber-supported mixed Mo/W-carbide catalysts with varying Mo and W compositions using either temperature-programmed reduction (TPR) or carbothermal reduction (CR). Regardless of the synthesis method, all bimetallic catalysts (Mo:W bulk ratios of 1:3, 1:1, and 3:1) were mixed at the nanoscale, although the Mo/W ratio in individual nanoparticles varied from the expected bulk values. Moreover, the crystal structures of the produced phases and nanoparticle sizes differed depending on the synthesis method. When using the TPR method, a cubic carbide (MeC_1–*x*_) phase with 3–4 nm nanoparticles was obtained, while a hexagonal phase (Me_2_C) with 4–5 nm nanoparticles was found when using the CR method. The TPR-synthesized carbides exhibited higher activity for the hydrodeoxygenation of fatty acids, tentatively attributed to a combination of crystal structure and particle size.

## Introduction

Combining two metals in one catalyst can result in a synergetic catalytic performance with respect to activity, selectivity, and stability compared with monometallic catalysts.^1−5^ Prime examples include the addition of non-noble metals to noble metals such as Pt, Au, and Pd for use in hydrogenation,^3^ hydrodeoxygenation,^4^ Fischer–Tropsch synthesis,^5^ and aqueous-phase reforming.^6,7^ For instance, during the conversion of biobased feedstocks, the addition of Ni, Co, or Fe to Pt decreases the adsorption energy of CO and H on the Pt surface, which results in a 3-fold activity increase for aqueous-phase reforming of ethylene glycol.^6,7^

Due to the limited availability of noble metals, replacement materials have become highly sought after, with the benefit of many having more favorable properties, such as tungsten carbides and molybdenum carbides whose poison stability may surpass the current noble metals in use.^8,9^ Monometallic Mo and W carbides are often applied as catalysts for reactions that involve hydrogen activation,^10−12^ recently (bulk) bimetallic MoW carbide catalysts have attracted attention.^13−25^ For example, Tran et al. observed a synergistic effect between Mo and W carbides in the hydrodeoxygenation of guaiacol to hydrocarbons.^19^ Mixed Mo/W-carbide catalysts exhibited superior catalytic activity due to the presence of H_2_-activating sites and oxophilic sites in the same catalyst. H_2_-activating sites were created by the interaction between Mo and W atoms while the presence of (metallic) W introduced oxophilicity. Mehdad and co-workers synthesized and characterized single-phase mixed MoW carbides with different Mo/W ratios for toluene hydrogenation.^22^ It was observed that an increasing W content led to decreasing activity relative to monometallic Mo carbides, but the selectivity toward the more desired products such as benzene and xylene increased. Wang et al. used a bimetallic carbide catalyst consisting of Mo_2_C and WC for the electrochemical hydrogen evolution reaction.^23^ Here they found that electrochemical activation can partially remove surface carbon that in turn changed the surface hydrophilicity, leading them to hypothesize that the contribution of the residual carbon protects the carbide from oxidation, maintaining high activity and stability of the catalyst.^24^

Though these examples highlight the relevance of Mo/W mixed carbides and potential synergistic effects, the relationship between synthesis history/path, catalyst characteristics, and catalytic performance of supported metal-carbides has not been fully investigated.^15,18,23,26^ Here we focus on the synthesis path for Mo/W mixed carbides to give handles to steer the performance of these type of catalysts.

For supported metal-carbides, two major synthesis routes have been developed, i.e., temperature-programmed reduction (TPR)^11^ and carbothermal reduction (CR).^27^ In the CR synthesis, the carbon support containing the metal precursor, e.g., ammonium heptamolybdate (AHM), is heated in an inert atmosphere to high temperature (up to 900 °C) where the carbon from the support reacts as reducing and carburization agent.^8,27−31^ The pathway from AHM to Mo-carbide proceeds in several steps, i.e., precursor decomposition, oxide formation, reduction, and carburization.^8,25,32−35^ In contrast to the CR synthesis of Mo-carbides, the synthesis of W carbides (from ammonium metatungstate (AMT)) is less studied,^28,36,37^ while no reports are available for the supported synthesis of mixed MoW carbides.

In TPR synthesis, often AHM or AMT, but the use of MoCl_5_/WCl_6_^17,38^ and ammonium tungsten oxide hydrate^15^ has been reported, is heated in a carbon containing atmosphere like CO,^39^ methane,^8,40,41^ or larger hydrocarbons.^42−44^ The TPR synthesis pathway of both W carbides and Mo carbides has been previously studied.^8,25,32−35^ As for the CR method also for TPR, the formation of the carbides from the precursors takes place via several steps, including precursor decomposition, oxide formation/reduction, and carburization.

To precisely synthesize catalysts understanding the reaction pathway from precursor to final catalyst is essential. For the synthesis of carbide catalyst temperature-programmed desorption coupled with mass spectrometry (TPD-MS), thermal gravimetric analysis (TGA), and X-ray diffraction (XRD) are indispensable tools. TPD-MS gives information on the evolved gases, such as the temperature at which CO evolves and the carburization temperature.^45,46^ Since TGA^20^ measures mass changes as a function of temperature, it is a suitable technique to follow the carburization process, especially for the CR method, while XRD^19^ gives information on the crystal structure during the formation of the oxides and carbides. In addition, scanning transmission electron microscopy coupled with electron diffraction (STEM-EDX^18^) provides information on the particle sizes and elemental distribution in the final material. Here we present a combination of these techniques in order to establish the reaction pathways during synthesis.

Though relevant insights into relationships between catalyst properties such as crystal structure,^47^ particle size,^48^ and catalytic performance for deoxygenations exist, a detailed understanding of supported bimetallic carbides is lacking, also noted by Mehdad et al. in 2019.^20^

Several studies have offered suggestions for the composition and structure of supported mixed carbides. Fu et al.^18^ synthesized a series of mixed MoW carbide catalysts supported on carbon nanotubes via carbothermal reduction, which was used for hydrogen evolution reactions. Based on XRD and TEM-EDS analysis, these researchers concluded that an orthorhombic MoW phase formed in which Mo and W were atomically mixed. Leclercq and co-workers^49,50^ used XPS analysis to explore the surface composition of bulk MoW materials and found Mo enrichment on the surface; however, they did not establish the exact composition and structure of the mixed MoW carbides.

In this paper, we report on carbon nanofiber-supported mixed MoW carbides synthesized via both the TPR and the CR methods. Combined TPD-MS, TPR-MS, TGA, XRD, and STEM-EDX will be used to investigate the influence of the synthesis method on the physicochemical characteristics of these catalysts. Hydrodeoxygenation of stearic acid will be used to relate the characteristics of the catalysts especially to their particle size and crystal phase to their catalytic activity.

## Material and Methods

### Carbide Catalyst Synthesis

Carbon nanofibers (CNF) were grown from a mixture of hydrogen (102 mL/min), nitrogen (450 mL/min), and carbon monoxide (260 mL/min) at 550 °C and 3 barg for 24 h over a reduced 5 wt % Ni/SiO_2_ catalyst (3 g), as reported previously.^51^ To remove SiO_2_ after growth, the mixture (CNF + Ni + SiO_2_) was refluxed three times in 1 M KOH for 1 h with intermediate decanting, and washing with 1 M KOH. After a final wash with water, the CNF were treated by refluxing in 65% concentrated nitric acid for 1.5 h to remove the remaining Ni and to introduce oxygen groups on the CNF surface. Finally, the CNF were washed with demineralized water to neutral pH and ground to a 90–120 μm fraction. BET surface area = 194 m^2^/g, total pore volume = 0.4 mL/g.

The CNF-supported catalysts were synthesized by incipient wetness impregnation (pore volume support 0.7 mL/g). Aqueous solutions of ammonium heptamolybdate (AHM; Sigma-Aldrich, 99.98% trace metals basis, pH 5), ammonium metatungstate (AMT; Sigma-Aldrich, 99.98% trace metals basis, pH 3), or a mixture of both salts (molar ratio of Mo:W = 1:3, 1:1, and 3:1) were used. All catalysts contained the same total metal loading of 0.9 mmol_metal_/g_catalyst_. After impregnation, the catalysts were dried overnight at 110 °C in static air and stored for further use.

The impregnated catalyst precursors (impregnated CNF) were carburized either via the temperature-programmed reduction method or the carbothermal reduction method in a tubular plug reactor. In the TPR method, the precursor (250 mg) was exposed to 20% CH_4_/H_2_ (total flow of 100 mL/min) for 2 h at 650 °C (β = 5 °C/min). For the carbothermal reduction, the samples were carburized in a N_2_ flow (50 mL/min) and heated from room temperature (RT) to 900 °C (β = 5 °C/min). To avoid contact with air, the carburized catalysts were directly transferred from the carburization reactor to a (N_2_ atmosphere) glovebox without exposure to air. For the 1:1 samples, some of the textural properties are BET surface area = 114 m^2^/g, total pore volume = 0.3 mL/g, BET = 151 m^2^/g, and total pore volume = 0.3 mL/g for CR and TPR prepared samples, respectively.

### Characterization

Temperature-programmed desorption (TPD-MS) and temperature-programmed reduction (TPR-MS) coupled with mass spectrometry were performed with a Micromeritics AutoChem II 2920 coupled to a Pfeiffer Vacuum ThermoStar mass spectrometer. For CR synthesis, the precursors (100 mg) were heated to 900 °C at 10 °C/min under helium (total flow of 20 mL/min). For TPR synthesis, the samples (100 mg) were exposed to 20% CH_4_/H_2_ (total flow of 100 mL/min) while heating to 750 °C at 5 °C/min.

Thermogravimetric analysis (TGA) was performed in 150 μL alumina crucibles (Mettler-Toledo) using a Mettler-Toledo TGA/DSC 1 apparatus. For CR synthesis, the precursors (40 mg) were heated to 1000 °C at 10 °C/min under nitrogen (100 mL/min). For TPR synthesis, the precursors (40 mg) were exposed to 20% CH_4_/H_2_ (total flow of 100 mL/min) while heating to 750 °C at 5 °C/min.

HAADF-STEM coupled with EDX was performed using an FEI Talos F200X transmission electron microscope operating in scanning transmission mode at 200 kV. The microscope is equipped with a high-brightness field emission gun (X-FEG) and a Super-X G2 EDX detector. The samples were ground and drop-casted from an ethanolic dispersion onto a lacey carbon-coated 300 mesh copper grid. Image analysis was performed with the Velox software..

XRD diffractograms were recorded on a Bruker D8 Advance equipped with an Lynxeye-XE-T PSD detector while using Cu Kα_1,2_ radiation (λ = 1.542 Å). The measurements were taken in the 2θ range of 20° to 80° with a step size of 0.05° at 1 s^–1^.

N_2_ physisorption isotherms were recorded using a Micromeritics, Tristar II Plus at liquid nitrogen temperature (−195.8 °C). 100 mg of sample was degassed at 200 °C for 2 h using a Micromeritics VacPrep 061. The pore volume and surface area of the samples were determined using the BET theory.

### Hydrodeoxygenation

Hydrodeoxygenation (HDO) of stearic acid reaction was performed in a 100 mL stainless-steel Parr 4598 Micro Batch stirred reactor system. Typically, 250 mg of catalyst, 2 g of stearic acid (Sigma-Aldrich, ≥95%, FCC, FG), 1 g of tetradecane as internal standard (Sigma-Aldrich, ≥99%), and 50 mL of dodecane (Sigma-Aldrich, ReagentPlus, ≥99%) were loaded in the reactor. Next, the loaded reactor was twice purged with 30 bar Ar and afterward flushed with H_2_. Subsequently, the reactor was pressurized to 30 bar H_2_, heated to 350 °C while stirring at 800 rpm. The reaction was performed for 4 h. Liquid samples from the reactor were taken at regular time intervals to investigate the product distribution. For analysis by gas chromatography (FID detection), the sample take from the reactor was split in two. Both samples were diluted with CH_2_Cl_2_:MeOH (2:1 v/v%) and analyzed. The average results of those two samples are reported, and the deviation from the average is indicated by error bars in the relevant graphs.

Recycles experiments were performed to assess the stability of the catalysts. After performing a catalytic experiment, the remaining reaction medium was decanted, and then the catalysts were washed/decanted with 100 mL of dodecane before adding fresh reactants and restarting the reaction as described above.

## Results and Discussion

### Investigation of the Carburization Pathway

First we investigated the effect of the metal compositions (Mo/CNF, W/CNF, and coimpregnated mixed MoW/CNF) on the synthesis pathway using two synthesis methods; the carbothermal reduction (CR) and temperature-programmed reaction (TPR). Synthesis of the carbides was followed by in situ gas phase analysis using TPD-MS and using the mass changes as inferred from TGA, complemented by the ex situ analysis of the crystal phases via XRD. As a control, the bare CNF support and a physical mixture containing equal parts of the two monometallic samples (Mo/CNF and W/CNF) is included. The two synthesis methods will be discussed separately for clarity starting with the results of the CR method.

#### Carbothermal Reduction (CR) Synthesis

**Figure 1:**
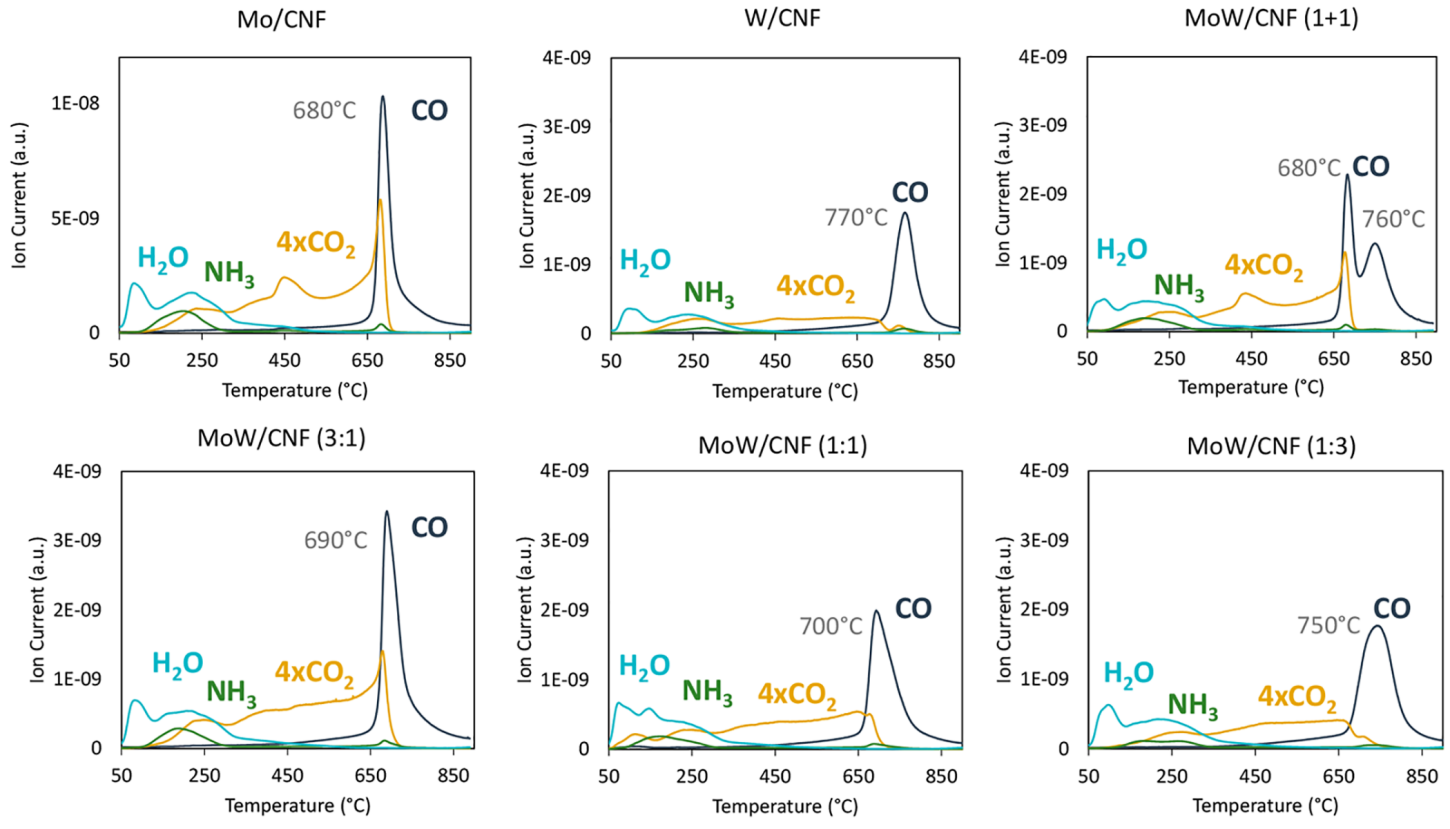
TPD-MS results of the carbothermal reduction of AHM (Mo/CNF), AMT (W/CNF), and a mixture of AHM and AMT (MoW/CNF) supported on carbon nanofibers heated to 900 °C (5 °C/min) under N_2_.

Figure 1 shows the result of the TPD-MS analysis conducted by heating the impregnated samples under inert gas (N_2_). The evolution of CO (*m*/*z* = 28), H_2_O (*m*/*z* = 18), NH_3_ (*m*/*z* = 16), and CO_2_ (*m*/*z* = 44, ×4) are displayed as a function of temperature.

All samples show a H_2_O peak at 100 °C and coinciding H_2_O and NH_3_ peaks at 250 °C. For Mo/CNF, a clear CO_2_ peak is also visible at 450 °C and again at 680 °C. These same peaks can also be identified for the physical mixture, but not in the monometallic W/CNF sample. The graphs for the bimetallic catalysts (MoW/CNF) show an increasing release of CO_2_ with increasing temperature, but without the discrete peak as seen for the Mo sample. The main feature for all samples is the large amount of CO evolved at temperatures above 650 °C. For the Mo/CNF, this CO release occurs at 680 °C, while for W/CNF it takes place at 770 °C. The CO peak of the Mo sample is sharper than that of the W/CNF sample, indicating a faster transformation. The CO evolution of the bimetallic catalyst occurs at a temperature that lies between the temperatures for each of the monometallic carbides (namely, at 690, 700, and 750 °C for a Mo:W ratio of 3:1, 1:1, and 1:3, respectively). This is in contrast with what can be seen for the physical mixture, namely, two distinctly separate CO peaks at 680 and 760 °C, matching the single peaks observed for the monometallic Mo and W samples, respectively. Together with the CO, the simultaneous evolution of some CO_2_ is observed for all samples except for the monometallic W sample.

**Figure 2:**
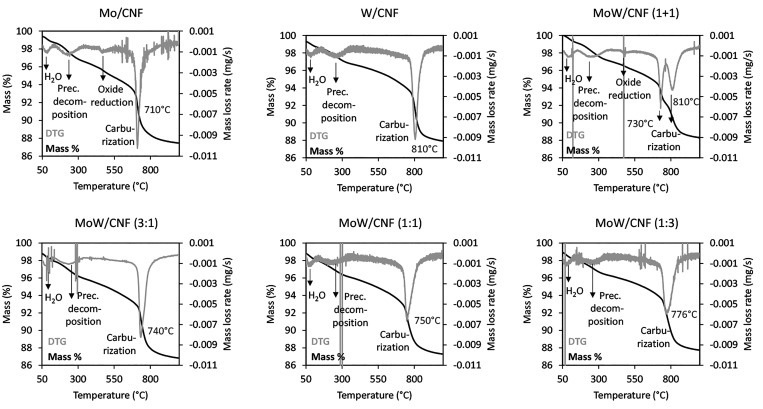
Thermogravimetric analysis showing the normalized mass loss and the mass loss rate (DTG) as a function of temperature for the carbothermal reduction of AHM (Mo/CNF), AMT (W/CNF), and a mixture of AHM and AMT (MoW/CNF) supported on carbon nanofibers under inert gas, heated up to 1000 °C.

In addition, TGA was used to study the CR process, Figure 2 shows the mass loss (%) and the mass loss rate (DTG) that occurred during heating to 1000 °C under N_2_. The mass loss at various temperature ranges is also reported and compared to the calculated theoretical mass loss for the assumed reactions, summarized in Table S1. All of the catalysts initially show a similar mass loss profile; a mass loss of 0.7% at 100 °C, followed by a mass loss of ∼2% at 250 °C. At 400–550 °C, a mass loss of 1.4% and 1.0% is observed for the monometallic Mo sample and the physical mixture, respectively. The other catalysts do show a slow decrease in mass over this temperature range (∼0.7%), but without a discrete peak in the DTG. The main mass loss of about ∼5.4% occurred at 710 °C for the Mo/CNF while the main mass loss of about ∼7.1% occurred at 810 °C for the W/CNF carbide. The DTG peaks for the bimetallic carbides lie between those of the two monometallic carbides, whereas the physical mixture shows two peaks, at 730 and 810 °C, which match to the single peaks observed for the Mo/CNF and W/CNF samples, respectively.

**Figure 3:**
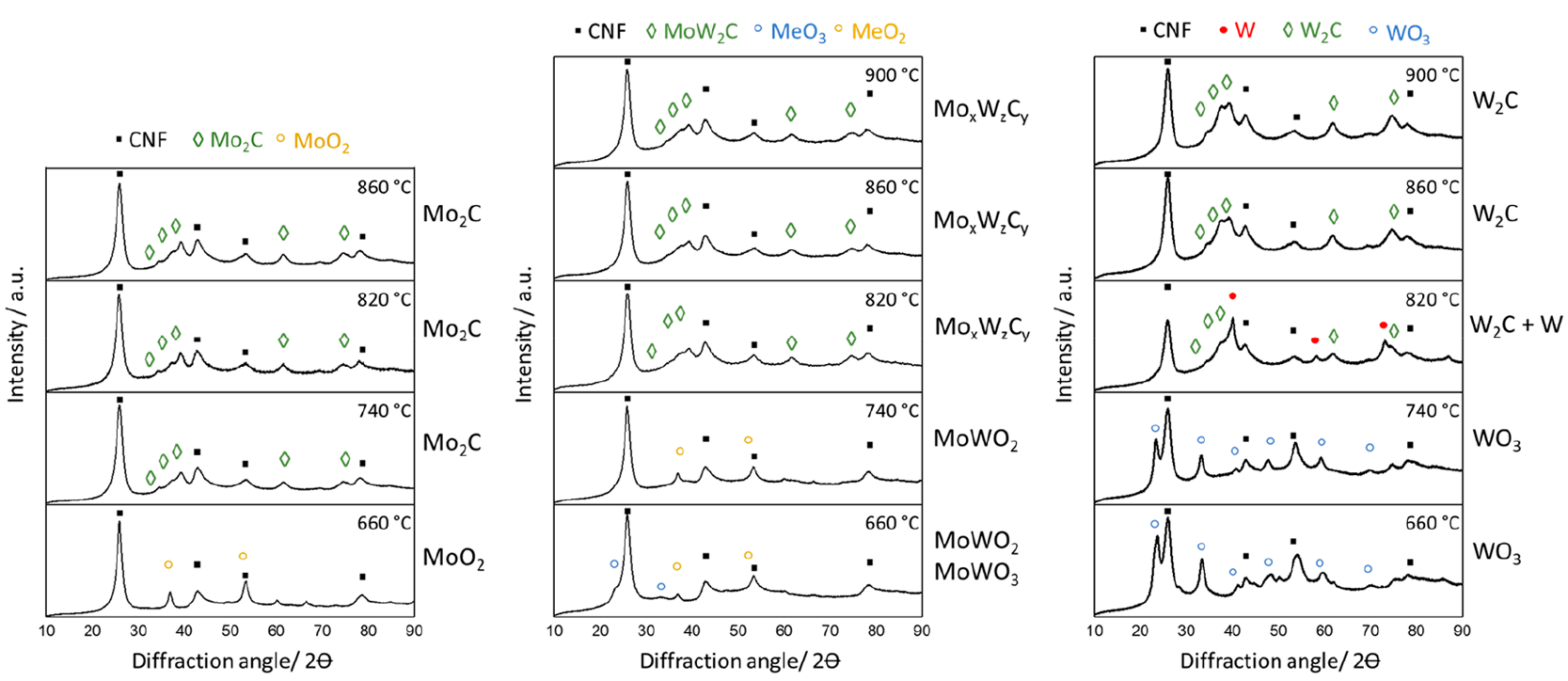
XRD of Mo/CNF (left), MoW/CNF (1:1, center), and W/CNF (right) after synthesis via the CR method at different temperatures.

Figure 3 displays the X-ray diffraction patterns of the two monometallic (Mo/CNF and W/CNF) carbides and the MoW/CNF (1:1) bimetallic carbide measured ex situ after synthesis up to different temperatures. The signals at 2θ of 28° and 43° belonging to the (002) and (101) reflections of the graphitic CNF^12^ are present in all patterns. At 660 °C, the Mo/CNF sample shows diffraction lines at 2θ values of 36.8°, 49.4°, 53.3°, 60.2°, 66.2°, and 78.6°, characteristic for the tetragonal MoO_2_ phase, while the W/CNF shows reflections at a 2θ of 23.6° and 33.3°, corresponding to the cubic WO_3_ phase. The pattern of the bimetallic sample (MoW/CNF) treated at 660 °C shows reflections that fit that of the monometallic dioxide and the trioxide phase (2θ of 23.6°, 33.2°, 36.9° and 53.3 2θ). Note that the Mo and W dioxide as well as the trioxide patterns have near identical diffraction patterns. At 740 °C, the reflections of the cubic trioxide have disappeared, and only the reflections of tetragonal dioxide are observed. When the temperature is increased, further diffractions at 34°, 37.5°, 39°, 61.5°, and 74.5° (2θ), characteristic for the hexagonal MeC_2_ phase (Me = W and/or Mo), appear for all the catalysts. The transformation of the MoO_2_ to the Mo_2_C occurs at 660 to 740 °C, while for the W/CNF and bimetallic MoW/CNF samples, this transformation starts between 740 and 820 °C. For W/CNF, we also observed an additional reflection (at a 2θ of 40.1°) together with the W_2_C, which can be assigned to cubic W carbide, this phase disappeared again after heating the sample to 860 °C.

**Scheme 1:**
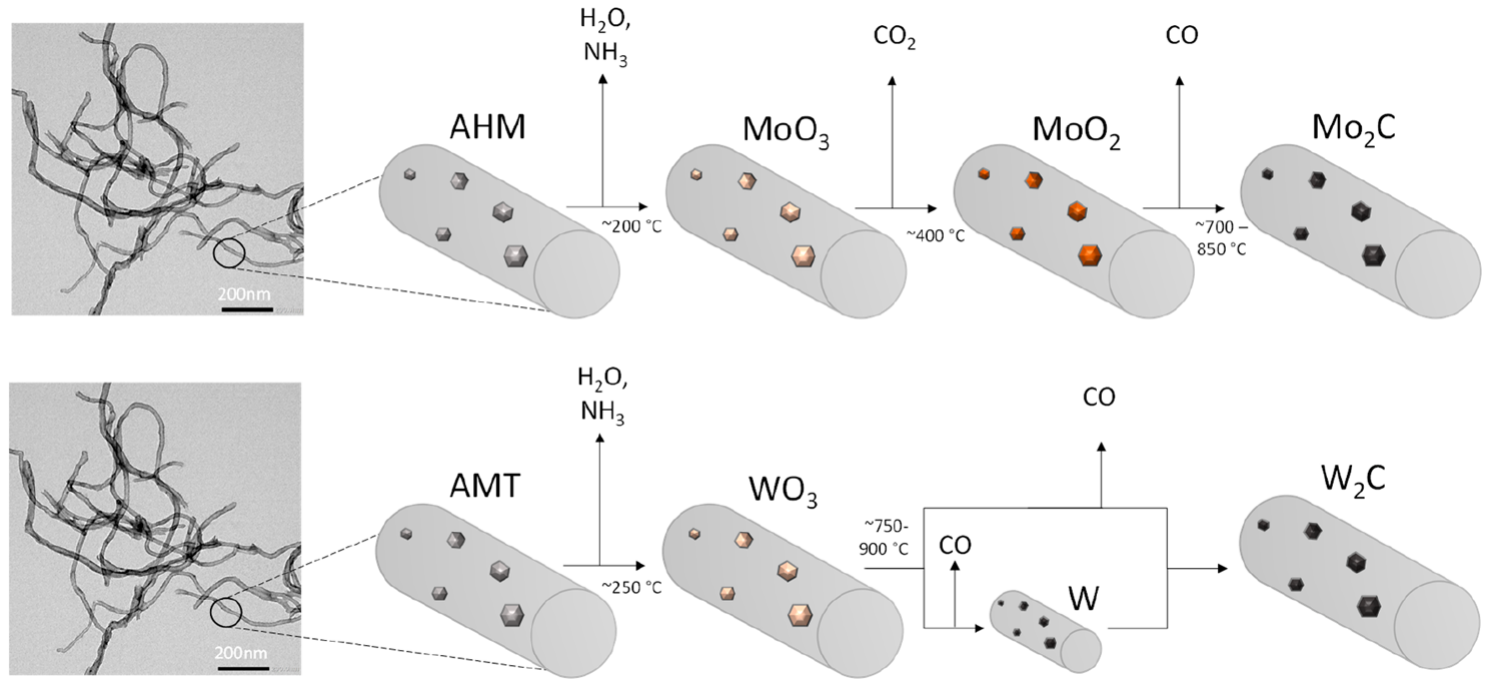
Proposed General Pathway for the Carbothermal Reduction (CR) of CNF Impregnated Ammonium Heptamolybdate (AHM) and Ammonium Metatungstate (ATM) The TEM image on the left shows pure CNF. The mixed carbides follow the AMH pathway.

Based on these results, we propose a synthesis with the CR method that follows the pathway displayed in Scheme 1. The first step during the CR synthesis is the release of absorbed water at 100 °C, which is observed with TGA (mass change of ∼1%) and TPD-MS (H_2_O evolution). Next, the breakdown of the ammonium heptamolybdate (AHM) and the ammonium metatungstate (AMT) precursor complex at 250 °C is correlated to the evolution of NH_3_ and H_2_O, with the precursor being transformed into its trioxide form (MeO_3_). These two steps were observed for the monometallic samples as well as for the bimetallic systems at similar temperatures. For the Mo/CNF sample, the theoretical mass loss during the precursor decomposition is 2.7% (see Table S1), which is somewhat higher than the measured value of 2.0%, indicating that the precursor hydration level is lower than assumed. For the W sample, 1.6% was calculated for the transformation which agrees well with the measured mass loss of 1.7%.

From previous studies, it is known that after the formation of the MoO_3_, the oxide can be reduced to its MoO_2_ form before carburization.^27,28,38,45,46,52^ We observed a CO_2_ peak at ∼450 °C and a significant mass change of 2.0% for the Mo carbide (and for the physical mixture), which we attribute to the reduction of MoO_3_ to MoO_2_. This mass change is in reasonable agreement with the calculated values of 1.8% for Mo. The simultaneously recorded heat flow revealed that this step was indeed exothermic (see Figure S1). In contrast with what we found for Mo/CNF, we detected neither a significant CO_2_ peak nor a mass change for the W/CNF sample in this temperature range. These observations are also in line with the XRD data, which confirmed the presence of MoO_2_ (at 660 °C) for Mo/CNF, but did not show any reflections attributable to WO_2_ for W/CNF. It appears that a (partial) reduction of MeO_3_ to MeO_2_ does takes place for the bimetallic systems (MoW/CNF), since at 660 °C, both MeO_3_ and MeO_2_ reflections were detected for the bimetallic system (1:1). Furthermore, in comparison with the W sample, the CO_2_ evolution at 450 °C (indicating the reduction to MeO_2_) is much greater for the bimetallic carbides. However, this formation appears to be too slow to observe with TGA, since no notable peak in the DTG was observed.

The final step of the carbide synthesis is the reduction of the MoO_2_ or WO_3_ to Mo_2_C and W_2_C, respectively. The carbide formation occurs in a temperature range of 610 to 900 °C and results in the release of CO (and to a lesser degree, CO_2_) detected by TPD-MS. This corresponds to the main mass loss step measured with TGA, originating from the reaction of carbon from the support with the metal oxide (see Table S1). The carburization of Mo/CNF occurs at 680 °C (TPD-MS) with a measured mass loss of 5.4% and the carburization of W/CNF taking place at 770 °C (TPD-MS) with a mass loss of 7.1%. Slightly higher measured mass changes in comparison with the calculated values, 4.6% and 6.4% for Mo/CNF and W/CNF respectively, are expected due to the additional mass loss related to the decomposition of support oxygen groups (see Figure S2). However, the main part of the measured changes in mass and the observed CO release can now be attributed to the carburization step. This is also in agreement with our XRD data which shows that the transformation of MoO_2_ to Mo_2_C occurs between 660 and 740 °C, while the transformation of WO_3_ to W_2_C occurs between 740 to 820 °C. For W/CNF, the WO_3_ phase is transformed to metallic W and directly to W_2_C at 740 to 820 °C. Full carburization of the metallic W takes place between 860 and 900 °C. It has been suggested that the reduction of the WO_3_ proceeds in two simultaneous steps (see Scheme 1), one in which the WO_3_ is directly transformed into the W_2_C and another one in which the carbide is formed via the metallic carbide.^28,37^ This could also explain why the CO peak for the carburization of W is broader than for Mo. The carburization of the physical mixture (MoW/CNF (1 + 1)) clearly shows two separate steps for the CO evolution and mass loss whereas the bimetallic samples (MoW/CNF) carburize in a single step with CO release and mass loss occurring at temperatures in between the monometallic carbides. In the XRD results, we can identify the formation of a hexagonal carbide at 820 °C which may correspond to a mixed carbide phase (Mo_*x*_W_*y*_C_*z*_).

#### Temperature-Programmed Reaction (TPR) Synthesis

**Figure 4:**
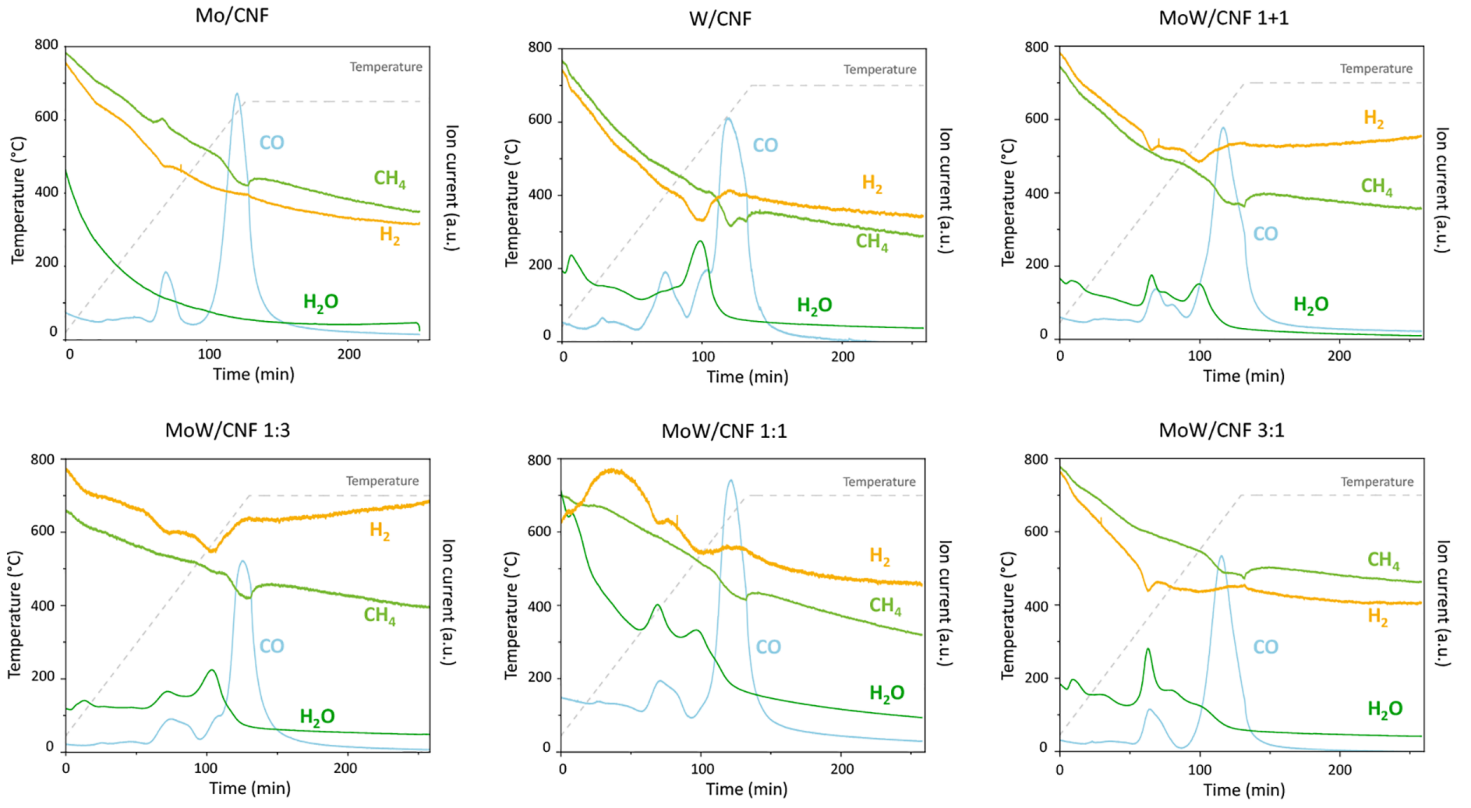
TPR-MS results of the temperature-programmed reaction of AHM (Mo/CNF), AMT (W/CNF), and a mixture of AHM and AMT (MoW/CNF) supported on carbon nanofibers heated to 650 °C (Mo/CNF) or 700 °C (others), 5 °C/min under 20% CH_4_/H_2_.

Figure 4 shows the result of the TPR-MS analysis conducted by heating the impregnated samples to a temperature of 650–700 °C (5 °C/min), followed by an isotherm of 2 h under 20% CH_4_/H_2_ (total flow of 50 mL/min). The temperature profile and evolution of H_2_ (*m*/*z* = 2), CO (*m*/*z* = 28), H_2_O (*m*/*z* = 18), and CH_4_ (*m*/*z* = 16) over time are displayed. CO_2_ evolution (not shown) also occurred but was in magnitude similar to the observed CO_2_ release for the bare CNF support (see Figure S2).

First, a broad release of H_2_O is visible over a large part of the temperature range with a first peak around 100 °C for most samples. Two prominent CO release peaks are present for Mo/CNF at 375 and 620 °C. For W/CNF these occur at 410 and 625 °C, here the main CO release peak has an additional shoulder around 520 °C. Contrary to the CR method, in TPR, reactive gases (CH_4_ and H_2_) are present that participate in the carburization process. Even though the CH_4_ and H_2_ signals show significant baseline drift, also here gas consumption and evolution peaks can be distinguished. For Mo/CNF, a H_2_ consumption peak occurs together with the CO release at 365 °C. Next a CH_4_ consumption peak is visible together with the main CO release peak and H_2_ evolution at 620 °C. For W/CNF, also a H_2_ consumption peak, together with CO and the main H_2_O release, is visible at a temperature of 520 °C. The data for W/CNF shows two separate CH_4_ consumption peaks, the first CH_4_ consumption peak occurs simultaneously with the release of CO at 625 °C, while the second one occurs at a slightly higher temperature of 680 °C. During the remainder of the isothermal dwell period, no further gases were released or consumed. The physical mixture (Mo + W/CNF (1 + 1)) and the bimetallic samples (MoW/CNF) show features that are a combination of the monometallic Mo/CNF and W/CNF samples.

**Figure 5:**
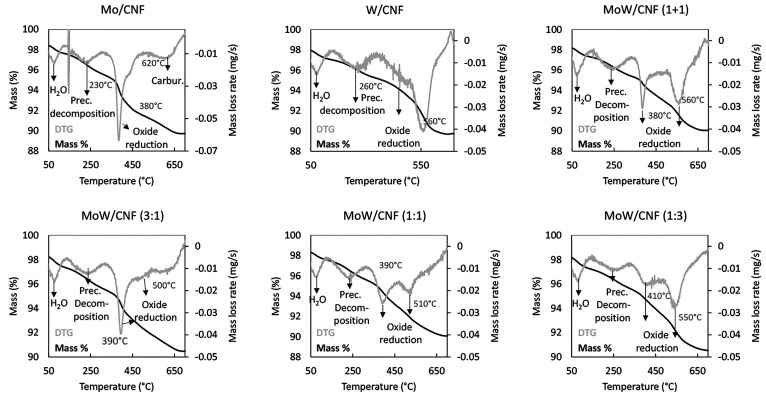
Thermogravimetric analysis showing the normalized mass loss and the mass loss rate (DTG) as a function of temperature for of the TPR of AHM (Mo/CNF), AMT (W/CNF), and a mixture of AHM and AMT (MoW/CNF) supported on carbon nanofibers under 20% CH_4_/H_2_ gas up to 700 °C.

TGA was also conducted during the TPR synthesis (20% CH_4_/H_2_, 700 °C), Figure 5 shows the mass loss (%) and the mass loss rate (DTG) for all catalysts. The measured mass losses for different temperature ranges are compiled in table S2 and compared to the theoretical values obtained for the assumed reactions. The first significant mass change (∼1%) was observed at ∼100 °C. At 150 to 300 °C, a broad peak in the mass loss rate of about ∼2% is present for all samples. A third significant mass loss of 2.2% occurred at 380 °C for the Mo/CNF sample while the next mass loss for W/CNF of about 1.8 wt % took place at 560 °C. For the physical mixture, two mass changes were observed at the same temperatures as found for the monometallic carbides (380 and 560 °C). Corresponding peaks were also observed in the mixed metal systems (MoW/CNF) at a slightly shifted positions, namely, 390 and 500 °C for the 1:3, at 390 and 510 °C for the 1:1 ratio, and at 410 and 550 °C for the 1:3. For Mo, an additional mass loss peak was detected at 620 °C, whereas the graph for W/CNF and the physical mixture even displays a minor mass gain at about 690 °C. For the bimetallic systems, only mass loss occurs in that temperature range, but no clear peak in the mass loss rate was observed.

**Figure 6:**
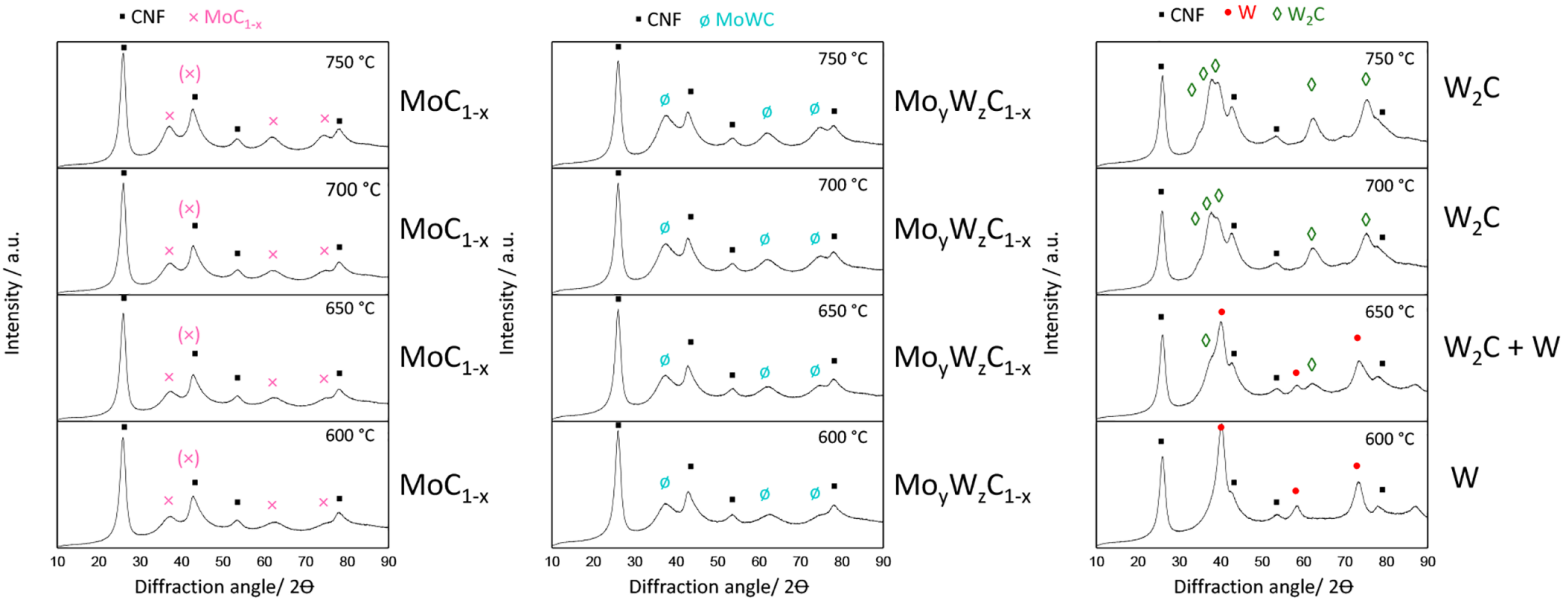
XRD of Mo_2_C/CNF (right), MoWC/CNF (1:1, center), and W_2_C/CNF after synthesis via the TPR method.

To gain further insight in the evolution of phases as a function of temperature XRD was used. Figure 6 shows the X-ray diffraction patterns of the two monometallic (Mo/CNF and W/CNF) carbides and the MoW/CNF (1:1) bimetallic carbide measured ex situ after synthesis up to different temperatures. The signals at a 2θ of 28° and 43° represent the (002) and (101) reflections of the graphitic CNF^12^ and are present in all patterns. At 600 to 750 °C, the Mo/CNF shows reflections at 37° and 67.5° 2θ, which can be attributed to the cubic α-MoC_1–*x*_ phase. For the W/CNF reflections belonging to the metallic cubic tungsten (40.1°) were the main phase observed at 600 °C. When the temperature was increased further to 650 °C the metallic W was partially transformed to the hexagonal W_2_C phase with reflections at 34°, 37.5°, 39°, 61.5°, and 74.5° (2θ). At 700 °C this transformation was complete and the hexagonal W_2_C was the only discernible phase. For the bimetallic system with the 1:1 Mo:W ratio, reflections representing a cubic α-MeC_1–*x*_ phase were observed.

**Scheme 2:**
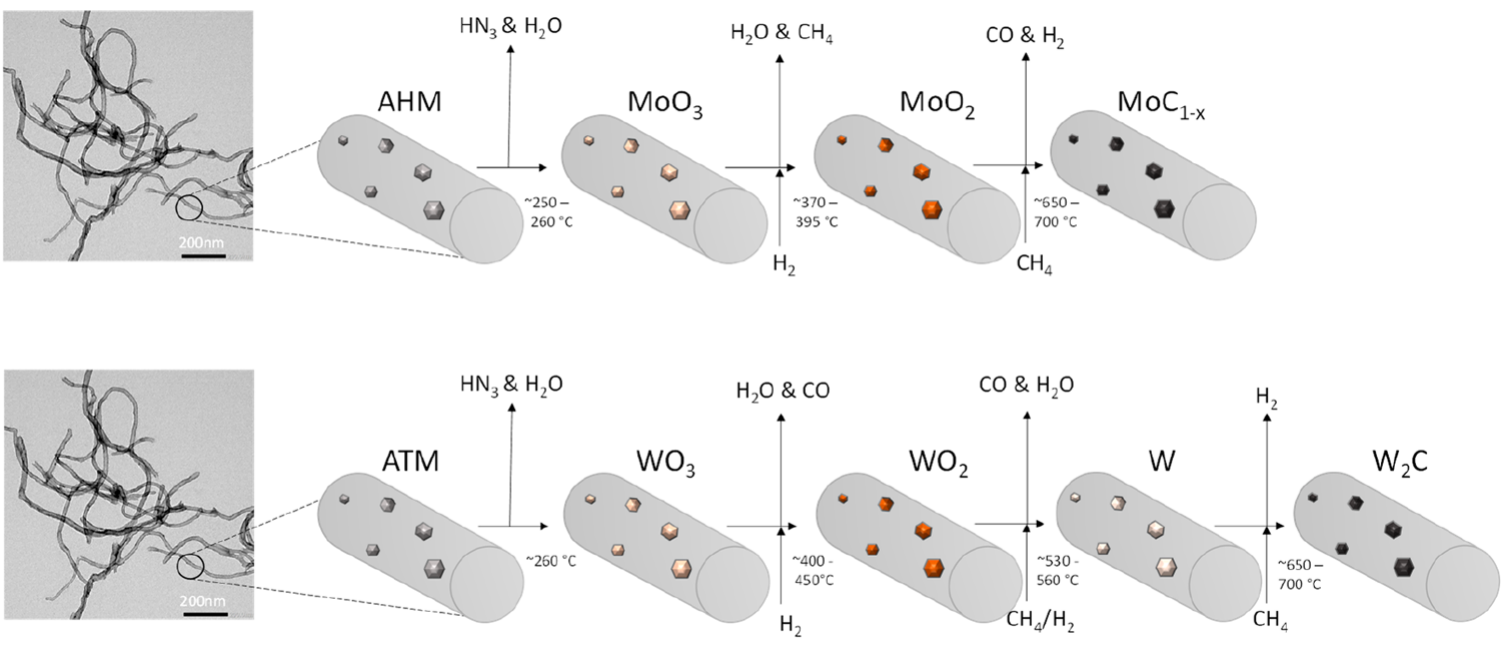
Proposed General Pathway for the Temperature Programmed Reaction (TPR) of CNF Impregnated Ammonium Heptamolybdate (AHM) and Ammonium Metatungstate (ATM) The TEM image on the left shows pure CNF. Mixed MoW/CNF follows the AHM pathway.

Based on these results we propose the synthesis with the TPR method follows the pathway displayed in Scheme 2. The initial mass loss at 100 °C can again (as for CR) be attributed to the evaporation of water absorbed on the CNF. This is supported by the evolution of H_2_O, as seen in the TPD-MS data. At 150 to 350 °C, the breakdown of the AHM and AMT precursor complex is correlated to the evolution of both H_2_O and ammonia (not shown) observed in TPR-MS. Here the salt is transformed into its respective trioxide form (MeO_3_). These two steps were observed for all samples at similar temperatures. For the precursor decomposition of W/CNF, the measured mass loss of 1.8% concurs with the theoretical mass loss of 1.6% for AMT (see Table S2). For Mo/CNF the observed mass loss of 2.1% is again lower than the theoretical loss of 2.6% for AHM likely because some dehydration already occurred, as was previously observed for the CR method.

The next step for the Mo/CNF is the simultaneous release of CO and the consumption of H_2_ with peaks in the TPD-MS at 370 °C, coinciding with a change in mass of 2.2% from the DTG peak at 380 °C. We attribute this to the reduction of the MoO_3_ to MoO_2_. This reduction can use both H_2_ and CH_4_ and has a theoretical mass loss of 1.30% (see Table S2). The larger observed mass loss suggests that some decomposition of support also occurs and/or part of the reduction could take place utilizing the carbon from the support, as was observed for the CR method. A final CO evolution peak is visible in the TPR-MS results together with a decrease in CH_4_ and an increase in H_2_ at 620 °C. At the same temperature, there is a broad corresponding DTG peak with a mass loss of 2.0%. This step is explained as the simultaneous reduction and carburization to form Mo_2_C (with a theoretical mass loss of 2.1%). The formation of Mo_2_C was already visible in XRD at 600 °C and no evidence of the formation of an intermediate metallic Mo phase was found in the temperature range of this transition.

For the W/CNF sample, after the precursor decomposition, we next observed H_2_ consumption in TPR-MS simultaneously with H_2_O and some CO release at 530–525 °C. After this the main CO release peak occurs at 630 °C simultaneous with H_2_ release and CH_4_ consumption. We interpret the first step as the reduction WO_3_ to WO_2_ utilizing mainly H_2_ as reductant. The second partially overlapping step is the reduction of WO_2_ to metallic W, corroborated by the XRD results showing metallic W as the dominant phase at 600 °C. This reduction primarily uses CH_4_ as a reductant since no H_2_O is released at this stage. These two steps match the main DTG peak at 560 °C which has a broad shoulder indicative of a two step process. The overall mass loss of 4.4% in the range 450–660 °C related to this peak agrees reasonably well with the theoretical mass loss of 3.6% for the overall reduction of WO_3_ to metallic W (see Table S2). Some additional mass loss is again expected due to the decomposition of oxygen groups on the support (see Figure S3). Finally a second CH_4_ consumption peak is visible at 690 °C at which point also a positive DTG signal is observed with a mass gain of 0.02%, which we attributed to the formation of the W_2_C carbide from the metallic W (with theoretical increase of 0.4%). This is again corroborated by the XRD results which show the transition from the metallic W to W_2_C is complete at 700 °C.

The physical mixture (MoW/CNF (1 + 1)) shows gas evolution and consumption as well as mass changes, at the same temperatures, as found for the individual monometallic carbides. For all mixed metal samples (MoW/CNF) also two DTG and two H_2_ consumption peaks close to the temperatures of the oxide reduction steps of the Mo/CNF and W/CNF are observed. This suggests that the oxide reduction of the bimetallic samples also takes place in two separate steps. However, for the bimetallic samples (MoW/CNF) these peaks are visible at slightly shifted positions. The second hydrogen release peak for these samples is less clearly defined because it transitions in a hydrogen release peak which again overlaps with CO release and CH_4_ consumption. For the bimetallic MoW/CNF sample a metallic phase was not detected with XRD and no mass gain attributed to carburization of metallic W or Mo was observed in TGA for any of the MoW/CNF samples. Therefore, it appears that, unlike the pure W/CNF, the bimetallic samples do not first reduce to a metallic phase before the carbide formation.

#### Effect of Metal Composition on the Carburization Process

After establishing the synthesis pathway for the monometallic (Mo/CNF and W/CNF) and bimetallic carbides (MoW/CNF) for the CR and TPR method, we will now discuss the effect of the metal composition on the carburization process.

**Figure 7:**
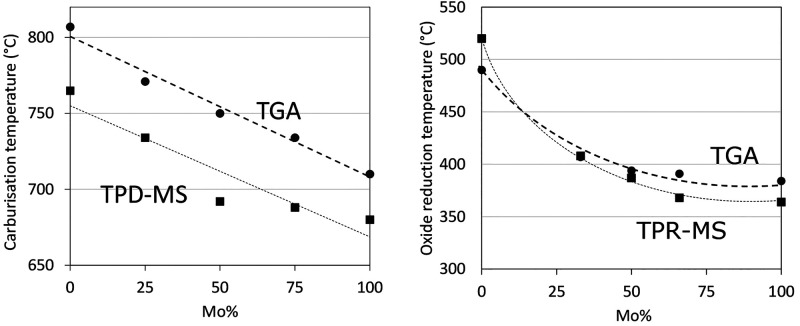
(a) Effect of metal composition expressed as Mo content (atom %) on the carbide formation temperatures in monometallic (W/CNF and Mo/CNF) and bimetallic (MoW/CNF, 3:1,1:1,1:3)) obtained from the mass loss (TGA) and CO release (TPD-MS). (b) Effect of metal composition expressed as Mo content (atom %) on the oxide reduction temperatures in monometallic (W/CNF and Mo/CNF) and bimetallic (MoW/CNF, 3:1,1:1,1:3)) obtained from the mass loss (TGA) and H_2_ consumption (TPR-MS).

In the carburization of the physical mixture, we observe two clear separate steps for the CO evolution in TPD-MS (Figure 1) and mass loss in TGA (Figure 2), which correspond to the independent formation of the individual Mo-carbide and W-carbide. For all bimetallic samples (MoW/CNF), on the other hand, the carburization of Mo and W appears to occur simultaneously in a single step. Figure 7a shows the effect of metal composition (represented by the percentage of Mo) on the carbide formation temperature obtained from both the CO release (TPD-MS) and mass change (TGA) during the carburization. The bimetallic systems carburize at a temperature in between that of the individual monometallic carbides and with increasing Mo content of the sample the carburization shifts to a lower temperature. The single-step carburization together with the systematic change in carburization temperature demonstrates that there is an interaction between the Mo and W in these samples and suggests that a mixed (bimetallic) carbide phase is formed. Note that these temperatures correspond to the peak maxima of the DTG and the CO release, representing the maximum observed rate of the transformation. However, the CO evolution peaks of the carburization (Figure 1) are broad and asymmetric, especially for samples with higher W contents. This suggests some heterogeneity with respect to size and/or composition of the nanoparticles. Clearly, the TPR-MS and TGA analyses in Figure 7a show the same trends for the carburization temperature, but the absolute temperatures are lower in the TPD analysis. We speculate that TPD-MS is performed in a plug-flow reactor while for TGA the gases are flown over the sample in a crucible, resulting in less intense contact between gas and catalyst particles. This, in turn, might result in a less effective heat and mass transfer, and as a consequence of differences in carburization temperature, fits with our observation that the difference is smaller at lower temperatures (Figure 7b).

For the TPR method we observed that the metal composition affects the oxide reduction temperatures. In the synthesis of the physical mixture, two separate steps for the H_2_ consumption in TPD-MS (Figure 4) and mass loss in TGA (Figure 5) are visible, corresponding to the independent formation of the individual MoO_2_ and WO_2_ phases observed for Mo/CNF and W/CNF. For all bimetallic samples (MoW/CNF), two separate steps are also observed. Figure 7b shows the effect of the metal composition (represented by the atomic Mo% fraction) on the temperature of the first oxide reduction step obtained from both the H_2_ consumption and mass loss steps, with both TPR-MS and TGA showing similar findings. Although very similar oxide formation temperatures are found for Mo/CNF and the mixed MoW/CNF 3:1, the reduction of the other bimetallic systems occurs at temperatures in between that of the individual monometallic carbides, and with increasing Mo content of the sample, the carburization shifts to a lower temperature. The change in oxidation temperature demonstrates that there is an interaction between the Mo and W in these samples and suggests that a mixed oxide phase is formed. Due to the second hydrogen release peak of the mixed metal samples (MoW/CNF) having an overlapping transition into a region of hydrogen release, the exact temperature for the second oxide reduction step is difficult to establish. However, as the bimetallic samples appear to oxidize in two steps could indicate that the heterogeneous phase with either a high W or Mo content is formed. For the TPR method, the actual carbide formation was visible in TGA as a mass gain for W/CNF and a mass decrease for Mo/CNF. For the mixed (MoW/CNF) samples, only mass loss occurred, however, a clear temperature for the carburization from these mass losses could not be obtained from TGA. The very broad peaks in TPR-MS for CO and H_2_ release and CH_4_ consumption associated with the carburization occur near the end of the temperature ramp and do not allow an accurate determination of the carburization temperature.

### Composition and Particle Size

The techniques described above indicate (indirectly) the formation of mixed phase carbides for the CR-prepared samples and mixed phase oxides for the TPR samples. To obtain additional insight on the potential interaction between W and Mo in the CR- and TPR-prepared metal carbides, we performed HAADF-STEM, EDX, and XRD analyses.

**Figure 8:**
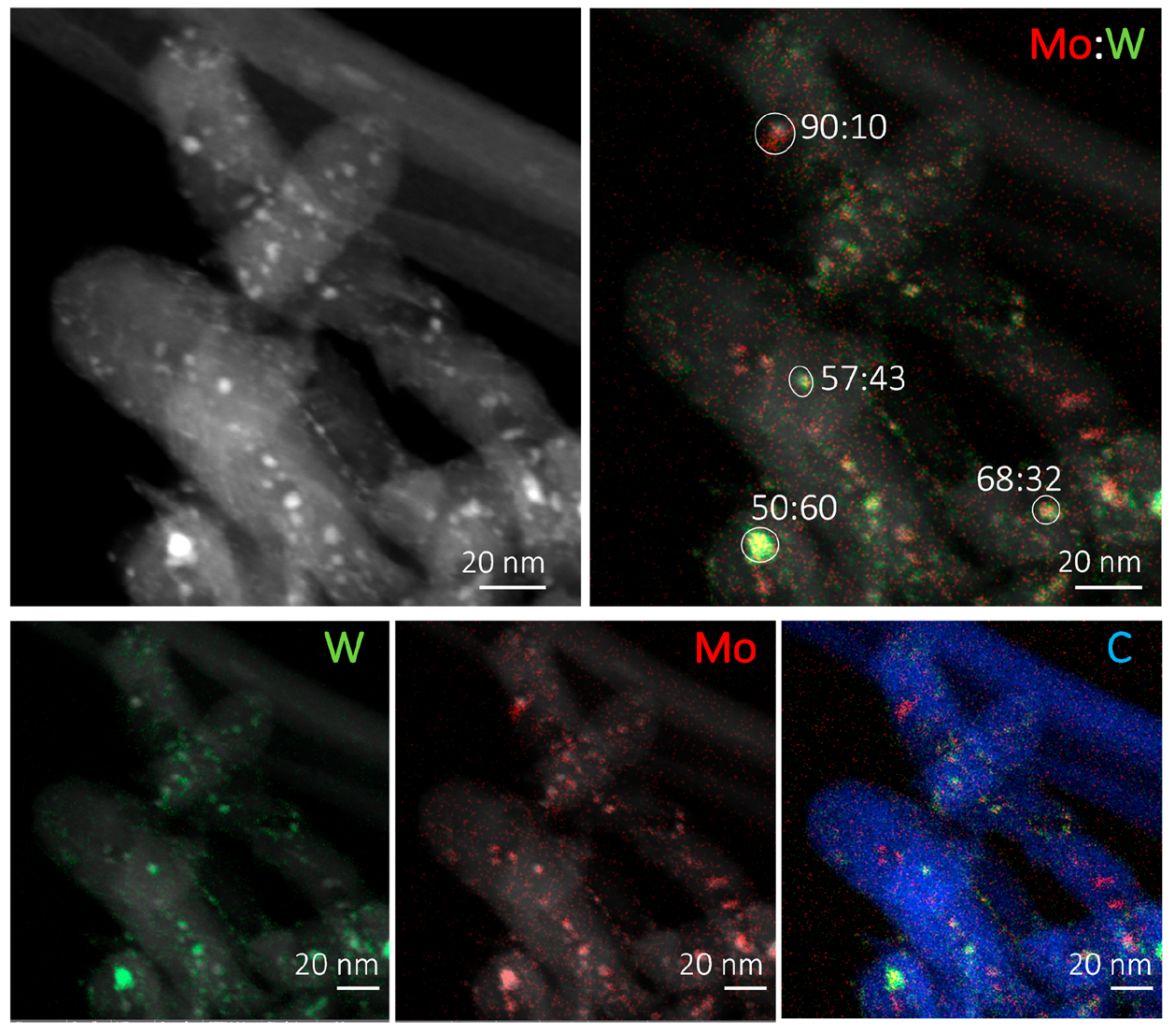
HAADF-STEM image with EDX map overlays the CR-prepared Mo/WCNF (1:1). The depicted ratios indicate the Mo:W ratio in the individual particles.

**Figure 9:**
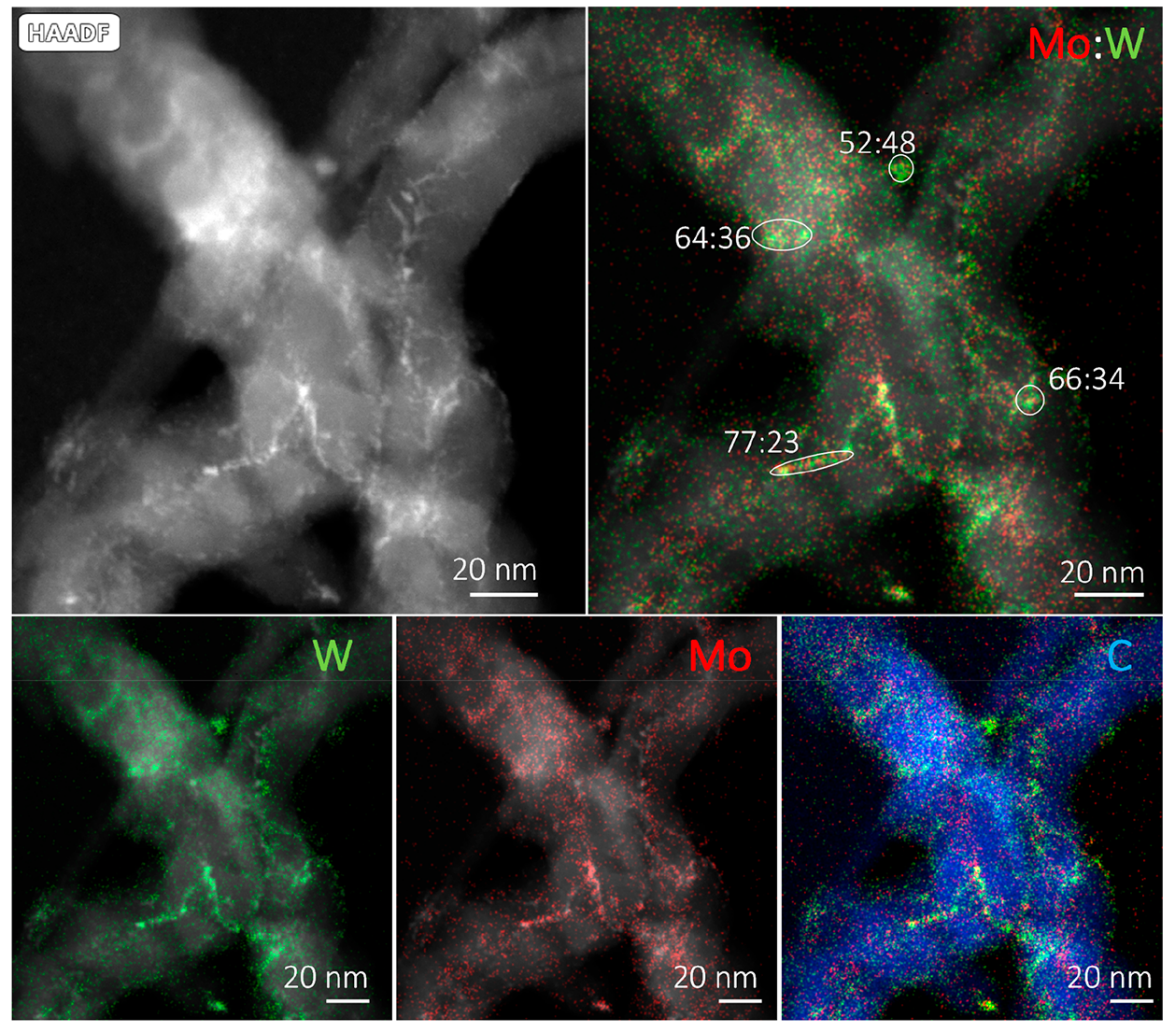
HAADF-STEM image with EDX map overlays the TPR-prepared Mo/WCNF (1:1). The depicted ratios indicate the Mo:W ratio in the individual particles.

Figures 8 and 9 show representative HAADF-STEM images and EDX images of CNF-supported MoW carbide samples prepared by CR and TPR (with Mo:W 1:1), respectively. The images and analysis for samples with the other two Mo:W ratios (1:3 and 3:1) can be found in the Supporting Information (Figures S4 and S5).

HAADF-STEM image clearly shows the metal-containing particles as bright spots on the CNF support. These particles have sizes of 4 to 5 nm. The elemental analysis indicates that W and Mo are well distributed over the support and analysis of this whole image yields an average Mo:W ratio of 55:45, which corresponds well to the bulk ratio of the prepared catalyst (i.e., 50:50 Mo:W). At the single-particle level, Mo:W ratios vary, showing that an inhomogeneous mixed phase had formed (e.g., Mo:W with 1:1 ratio shows ratios of 40:60, 57:43, 90:10, and 69:31). No monometallic particles were observed for any of the CR-synthesized samples.

The particle sizes are marginally smaller (3–4 nm) in comparison with samples prepared by the CR method (4–5 nm). Again, the Mo:W bulk ratio (50:50) is similar to the ratios in the whole image. The single-nanoparticle analysis yielded variable Mo:W ratios, which are distributed around the bulk ratio (52:48, 64:36, 77:23, and 66:34), similar to the sample prepared by the CR method, indicating the formation of a mixed metal carbide phase with some heterogeneity in composition.

From these HAADF-STEM and EDX analyses, we can conclude that a mixed phase formed on a single particle level, irrespective of the synthesis method. This is in agreement with the inferred interaction of Mo and W from the shift in carburization temperature obtained from the TPD-MS/TPR-MS and TGA results. Also, the variation in the Mo:W ratios of the individual nanoparticles found with EDX explains the broad peak observed during the TGA and TPD-MS analyses and the absence of two isolated carburization peaks representative of Mo and W.

**Figure 10:**
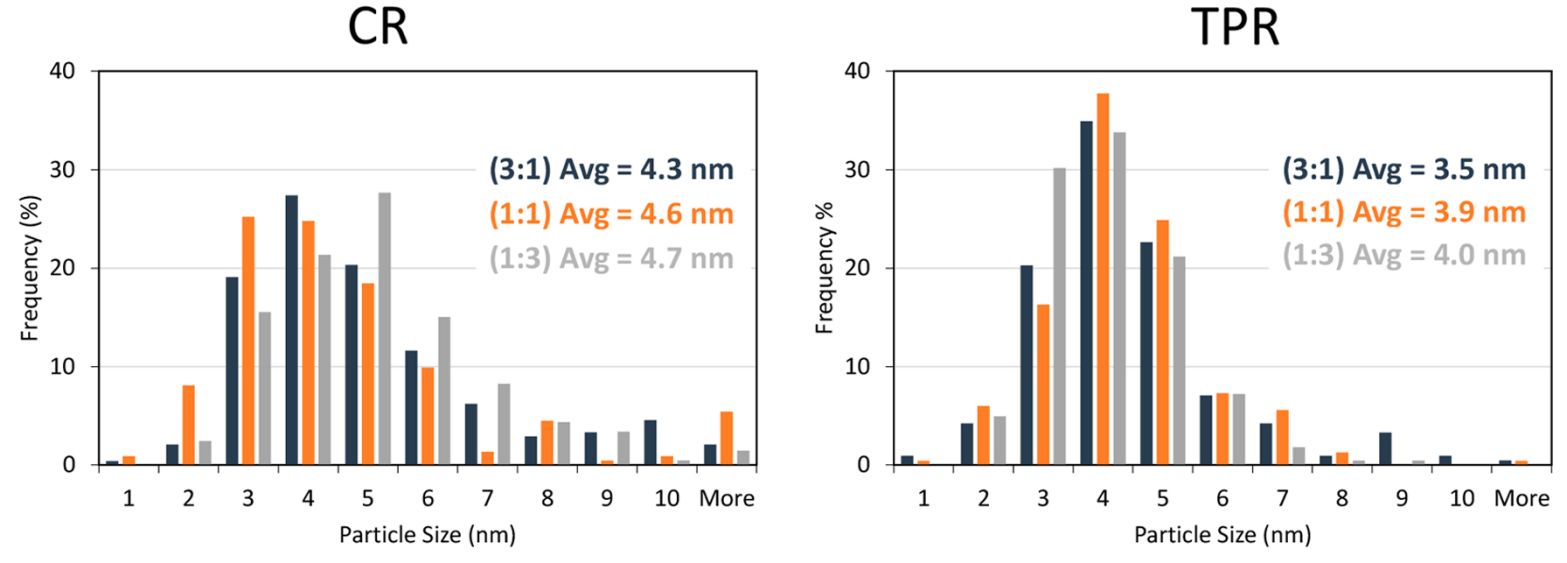
Particle size distribution of CR (left) and TPR (right) carburized bimetallic carbide samples.

In addition, we evaluated the particle size distribution of the mixed metal nanoparticles. Figure 10 presents the histograms of the particle size distribution of the bimetallic carbide samples carburized via the CR and the TPR. The average particle size for the TPR was 3–4 nm while the CR prepared samples have average particle size in tween 4–5 nm, this is the result of the broader size distribution of the CR samples compared to the TPR prepared samples.

**Figure 11:**
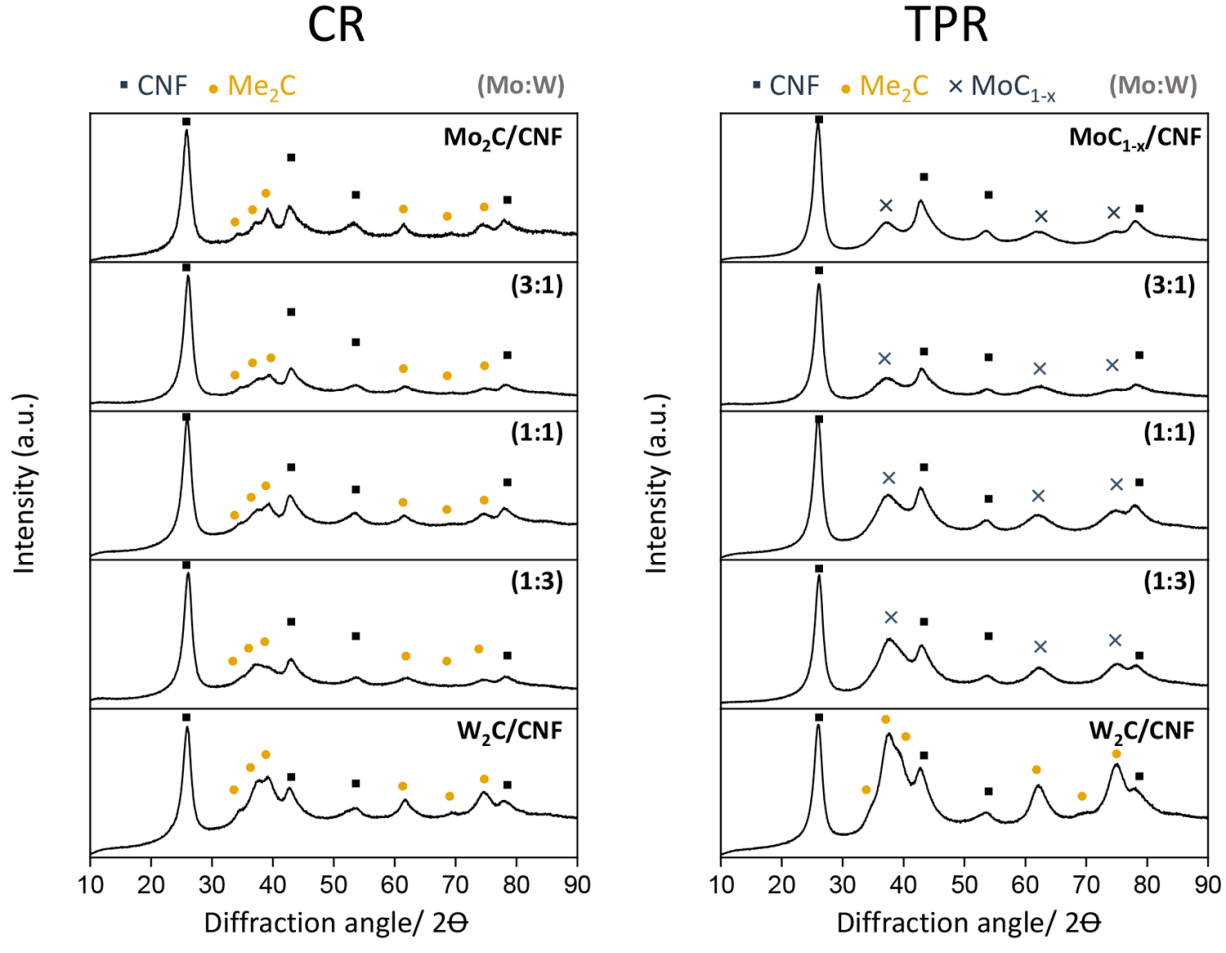
XRD patterns of monometallic carbide catalyst samples and bimetallic carbide catalyst samples, synthesized via TPR under 20% CH_4_/H_2_ (left) and via CR in N_2_ (right).

After establishing that synthesis via CR and TPR both resulted in mixed-metal particles (though with varying Mo:W ratios on single nanoparticles and marginally different particle sizes), we used XRD to establish which crystal phases have formed in these materials. Figure 11 shows the XRD patterns of all samples after the carburization. The signals at 2θ = 28° and 2θ = 43° represent the (002) and (101) reflections of the CNF.^12^ All XRD patterns of CR-prepared carbides show a similar pattern with diffractions at 2θ values of 34.4°, 37.7°, 39.4°, 61.5°, 69.2°, and 74.7°, indicated by the yellow squares, these reflections correspond to the hexagonal phase of β-Mo_2_C or β-W_2_C.^11,15,18,32,53,54^ It has previously been suggested that Vegard’s law^18^ can be used to prove the formation of a MoW mixed-metal carbides phase. We, however, did not observe a systematic shift in peak position with increasing Mo content. First, the difference between β-Mo_2_C and β-W_2_C is intrinsically small (0.1 2θ) and hence is difficult to observe for the very broad diffraction peaks for these mixed-metal nanoparticles. Second, for these overlapping broad reflections it is not possible to unambiguously ascribe a shift to either a change in *d*-spacing or the superposition of individual peaks (see Figure S6 (CR) and Figure S7 (TPR) in the Supporting Information for more details).

For the carbide catalysts prepared via TPR, different crystal structures were found for the Mo/CNF and W/CNF samples (Figure 11, right). The Mo/CNF sample prepared via TPR shows a broad reflection at a 2θ of 39°. This reflection represents the cubic α-MoC_1–*x*_ phase (less stable than the hexagonal form^55^), here indicated by the blue crosses.^8,56−58^ The W/CNF prepared via TPR displays reflections at 2θ values of 34.4°, 37.7°, and 39.4°, similar as seen for the CR prepared carbides. However, the peak at 37.7° is much sharper and higher in intensity, which makes it difficult to conclude that this carbide phase consists exclusively of a hexagonal W_2_C phase. The bimetallic carbides show a broad peak at a 2θ value of 39°, which can be attributed to the cubic carbide phase. However, since peaks are broad, it cannot be excluded that a minor hexagonal phase is also present.

### Catalytic Performance

The catalytic performance of the prepared catalysts was evaluated for stearic acid conversion in a batch reactor (350 °C and 30 bar H_2_), after 1 h. It should be noted that we found that above the used stirring rate of 800 rpm, the reaction rate became independent of the stirring rate, thus, H_2_ mass transfer did not play a significant role (see Figure S8). Furthermore, similar internal mass transfer properties are expected for all catalysts based on the similarity in porosity, as shown in Table S3.

**Figure 12:**
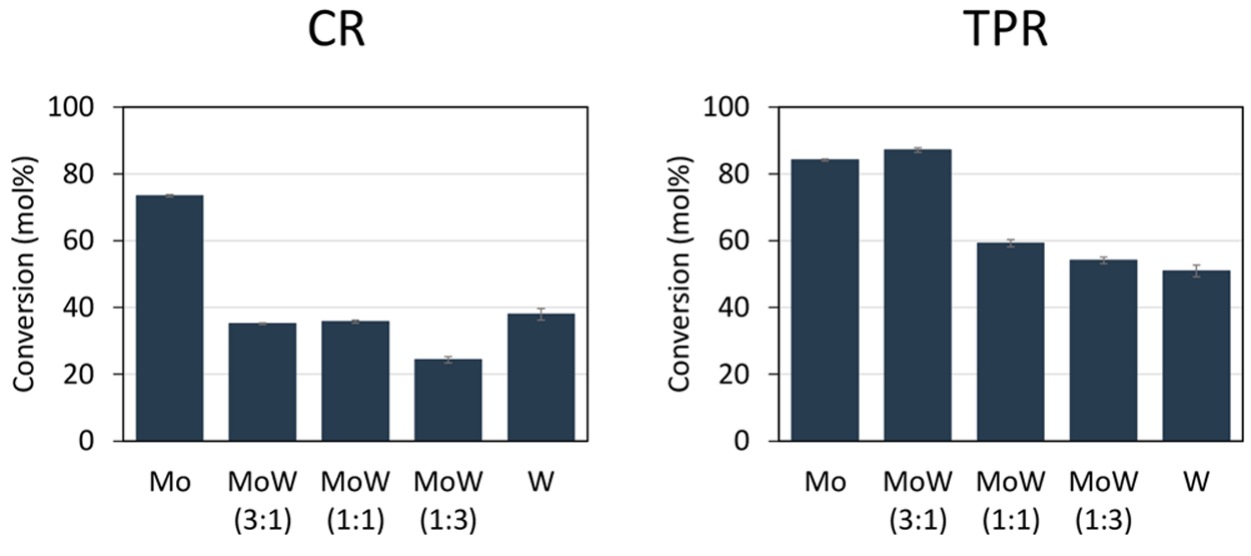
Stearic acid conversion over TPR-carburized (right) and CR-carburized (left) CNF-supported carbides after 1 h (250 mg of catalyst, 2 g of stearic acid, 350 °C, 50 mL of solvent, and 30 bar H_2_).

Figure 12 displays the activities of the CR and TPR samples as a function of composition. For the monometallic catalysts, the Mo carbide catalysts (75 (CR) or 85 (TPR) mol % conversion) exhibited superior activity compared to the W carbide catalysts (35 (CR) 45 (TPR) mol % conversion), irrespective of the synthesis method, in line with activities reported by Gosselink et al. and Stellwagen et al.^28,59^

Considering that bimetallic carbides are also active for fatty acid conversion, for the CR samples, their activity resembles that of W carbide, while for the TPR-synthesized mixed carbides, the activity is more between that of the monometallic catalysts. Unlike previous reports,^18,19^ we observed no enhanced catalytic activity for the bimetallic carbide catalysts. These results were found to be reproducible over repeated syntheses of these catalysts (see Supporting Information, Figure S9).

In general, the TPR-synthesized carbide catalysts with the mainly cubic crystal structure and with smaller nanoparticles (3–4 nm) provided faster hydrodeoxygenation (on a weight basis) than the CR-synthesized carbide catalysts with the hexagonal structure and larger nanoparticle size (4–5 nm). The CR-prepared samples converted ∼20–40 mol % of the stearic acid, while the TPR-prepared catalysts converted 50–85 mol %. It has previously been claimed for monometallic carbides that the cubic carbide phase is more active than the hexagonal phase.^8,35,56,60^ This is in line with our results for the bimetallic catalysts and could explain the difference in activity between samples synthesized via TPR and CR as well.

Since the particle sizes of the TPR samples are slightly smaller (3–4 nm) compared to those of the CR prepared samples (4–5 nm), part of the increase in activity of the TPR samples can also be explained by the higher carbide surface area in the TPR samples. However, the activity increased more than might be expected based on particle size, therefore, we conclude that the difference in crystal structure also plays a role; note that these particles are too large for intrinsic particle size effects.^28,61^

**Scheme 3:**
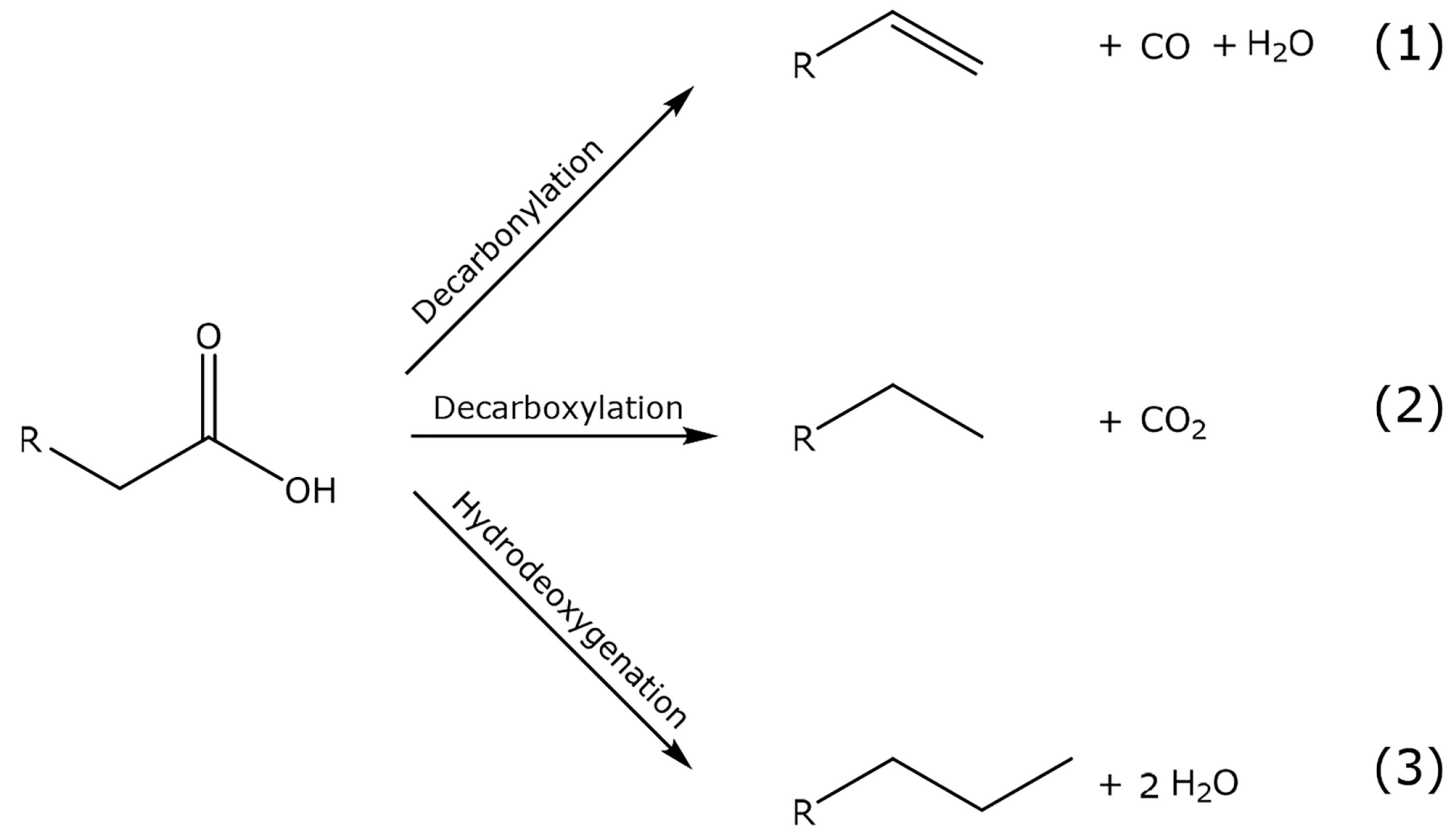
Possible Pathways for the Deoxygenation of Fatty Acids

**Figure 13:**
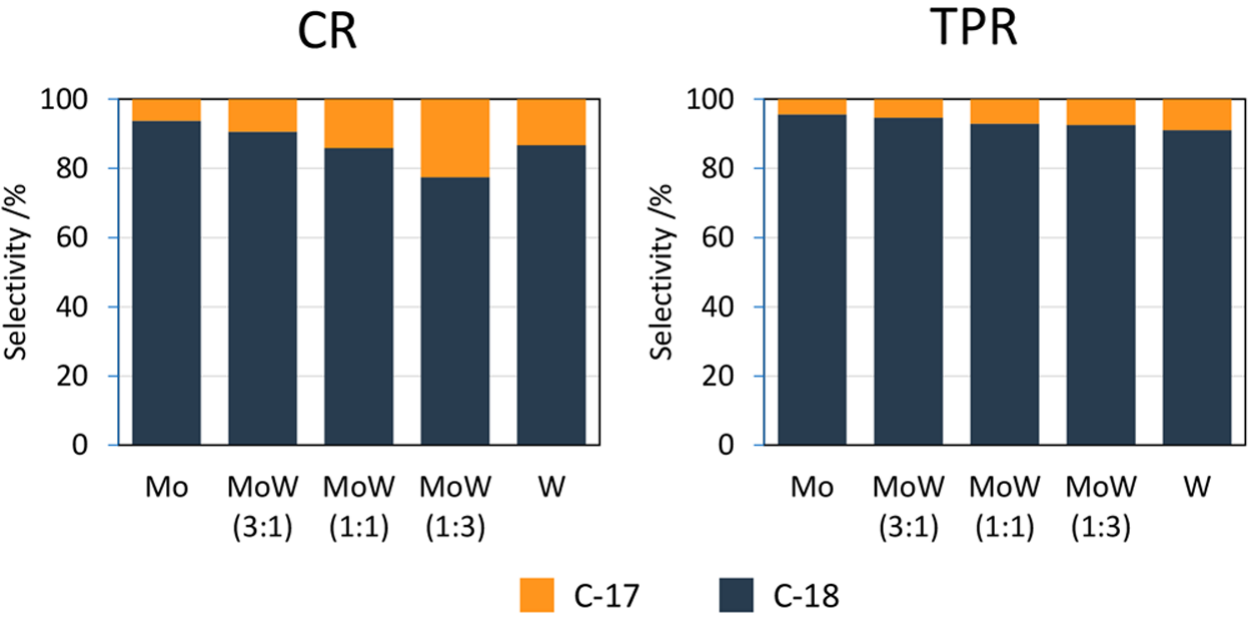
HDO and DCO selectivity of TPR-carburized (right) and CR-carburized (left) catalysts after 1 h (250 mg of catalyst, 2 g of stearic acid, 350 °C, 50 mL of solvent, and 30 bar H_2_).

Depending on reaction conditions and the catalysts, the deoxygenation of fatty acid can occur via different pathways (Scheme 3). The fatty acid can be decarbonylated and decarboxylated [DCO pathways (1) and (2)], yielding hydrocarbon chains with one carbon atom less compared to the reactant. Another possibility is the hydrodeoxygenation (HDO, pathway 3) of the fatty acid, resulting in a hydrocarbon with the same chain lengths as the reactant. The conversion of stearic acid over Pd, Pt,^62^ and Mo and W oxide^36^ catalysts goes primarily via the decarboxylation pathway and yields heptadecane. In contrast, the Mo and W carbides have previously been reported to convert stearic acid via the HDO route.^28,36,63,64^Figure 13 shows that the monometallic carbides and the mixed carbides mainly produce octadecane (C-18), suggesting that the majority of deoxygenation proceeds via the hydrodeoxygenation for all catalysts. Some C-17 products were also observed, and these can be produced via a direct decarboxylation or decarbonylation (DCO) of the stearic acid, indicating that some oxygen-containing species were present as catalyst.^28^ A direct comparison in selectivity between CR and TPR is not viable since the conversion levels for those catalysts were different. Nevertheless, the selectivity of the mixed carbides is similar to that of the monometallic carbides, indicating that similar active sites were formed in the bimetallic catalysts as in the monometallic carbides.

**Figure 14:**
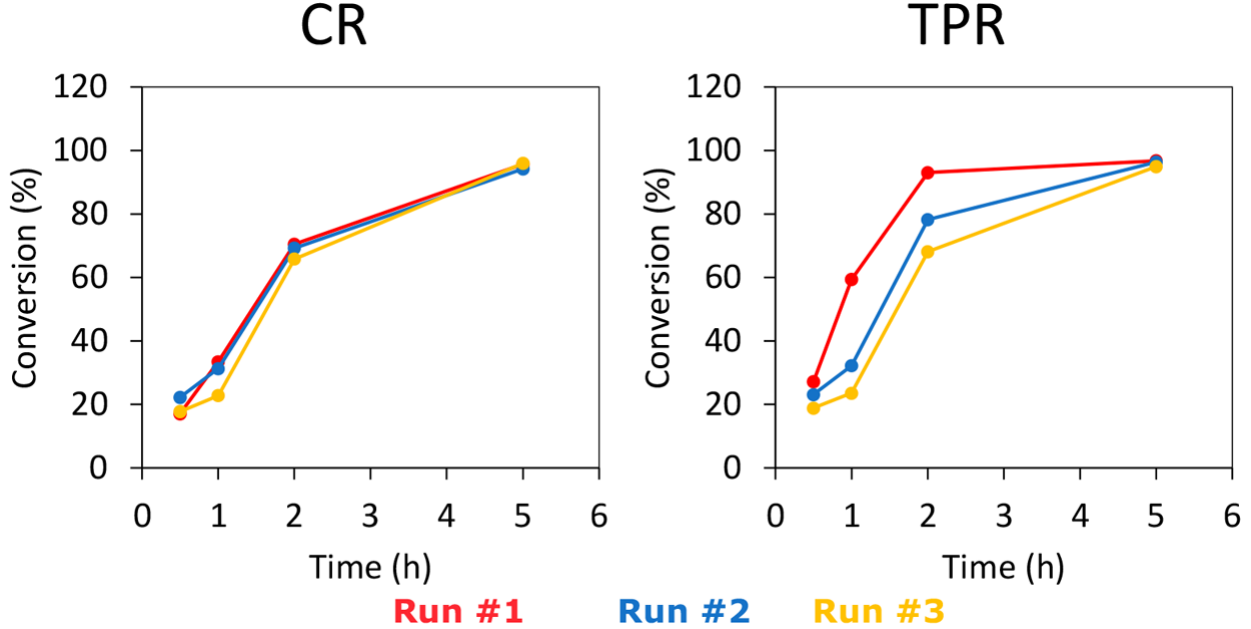
Multiple runs of stearic acid deoxygenation over MoW/CNF 1:1 (250 mg of catalyst with a metal loading of 0.9 mol/g_catalyst_, 2 g of stearic acid, 350 °C, 50 mL of solvent, 30 bar H_2_).

The catalyst stability was tested for the MoW/CNF (1:1) sample prepared via both the CR method and the TPR method. Subsequent experiments with one batch of carbide catalysts were conducted, with stearic acid conversions displayed in Figure 14. The results show that the CR-prepared catalysts remain stable for the 3 runs. In contrast, the activity of the TPR-prepared catalyst slowly drops after the second and third run. This indicates that the CR-prepared catalysts are more stable than the TPR-prepared catalysts.

## Conclusions

In this study, we demonstrated that a CNF-supported mixed-MoW carbide phase can be synthesized via the CR and TPR methods. TGA and TPD-MS analyses suggested the formation of the formation of truly mixed carbides which was confirmed by STEM-EDX analysis. However, the composition of the formed nanoparticles was not homogeneous though the overall composition matched the expected bulk values.

Irrespective of the sample composition and synthesis method, it is clear that the carbides prefer the hydrodeoxygenation pathway with a minor contribution (10%) from the decarbonylation/decarboxylation pathway. The catalytic activity of the mixed-metal carbides falls between that of the two monometallic catalysts, while the TPR-prepared samples were more active for the hydrodeoxygenation of fatty acids relative to CR-prepared samples. The higher activity of the TPR-prepared samples was related to the presence of the cubic carbide phase and partially to the smaller nanoparticle sizes (higher surface area).

## Supporting Information Available

Figure S1: Heat flow (HF) and mass loss of Mo/CNF in TGA for CR and TPR catalyst. Figure S2: TPD-MS and TGA data for bare CNF. Figure S3: TPR-MS of bare CNF under TPR conditions. Figure S4: STEM-EDX of CR pepared samples with 1:3 and 3:1 Mo:W ratios. Figure S5: STEM-EDX of TPR pepared samples with 1:3 and 3:1 Mo:W ratios. Figure S6: XRD patterns of CR carburized samples. Figure S7: XRD patterns of TPR carburized samples. Figure S8: Influence of stirring rate on conversion. Figure S9: Activity data with reproducibility margins. Table S1: Theoretical and measured mass losses during CR. Table S2: Theoretical and measured mass losses during TPR. Table S3: Pore areas of CR and TPR samples (PDF)

## Abbreviations

AHM: ammonium heptomolybdate
AMT: ammonium metatungstate
CNF: carbon nanofibers
CR: carbothermal reduction
DCO: decarbonylation and decarboxylation
DTG: derivative thermogravimetry
EDX: energy-dispersive X-ray spectroscopy
HAADF-STEM: high-angle annular dark-field scanning transmission electron microscopy
HDO: hydrodeoxygenation
FEG: field emission gun
FID: flame ionization detector
HER: hydrogen evolution reaction
STEM-EDX: scanning electron transmission electron microscopy energy-dispersive X-ray analysis
TEM-EDS: transmission electron microscopy energy-dispersive X-ray spectroscopy
TGA: thermogravimetric analysis
TPD-MS: temperature-programmed desorption mass spectrometry
TPR: temperature-programmed reduction
TPR-MS: temperature-programmed reduction mass spectrometry
XPS: X-ray photoelectron spectroscopy
XRD: X-ray diffraction
