# Supplementary material for: Synthesis and Characterization of Supported Mixed MoW Carbide Catalysts
Source: J Phys Chem C Nanomater Interfaces. 2023 Apr 17;127(16):7792–807. doi: 10.1021/acs.jpcc.2c08352 (PMC10150395; doi:10.1021/acs.jpcc.2c08352)
Supplement: Supplementary file 1 — jp2c08352_si_001.pdf [file jp2c08352_si_001.pdf]

Supplementary Information

Synthesis and Characterization of Supported Mixed MoW Carbide Catalysts

M. Führer[1], T. van Haasterecht[1], E.J.J. de Boed[2], P. E. de Jongh[2] and J. H. Bitter[1,*]

[1]*Biobased Chemistry and Technology, Wageningen University, PO Box 17, 6700 AA Wageningen, the Netherlands*

[2]*Materials Chemistry and Catalysis, Debye Institute for Nanomaterials Science, Utrecht University, Universiteitsweg 99, 3584 CG Utrecht, the Netherlands*

*To whom correspondence should be addressed. E-mail: harry.bitter@wur.nl

Contents

Thermal analysis 2

CR of the bare support material (CNF) 3

Mass loss during CR synthesis 4

TPR for the bare support material (CNF) 4

Mass loss during TPR synthesis 5

Element mapping (STEM-EDX) 5

N_2-physisoption 8

Evaluation of the XRD carbide reflections 9

Hydrogen mass transfer limitations 11

Reproducibility 12

References 12

Thermal analysis

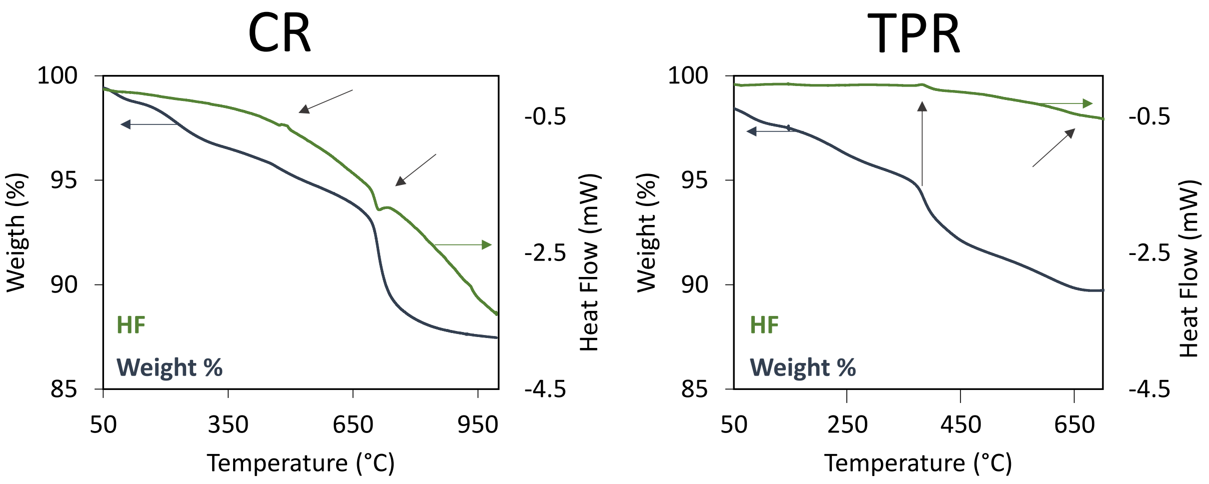

Figure S1. TGA-plots of Mo/CNF mass loss and heat flow of both carburization methods CR (left) and TPR (right).

To explore whether thermal effects could yield additional information on the carburization process, we also performed differential thermal analysis (DTA) simultaneously with TGA. Figure S1 shows the weight loss of the heat flow versus the temperature of the Mo carbide sample during the carburization process. On the left, the CR method is displayed and on the right, TPR. The positive heat flow indicates that the process was exothermic. As shown, the reduction of MoO_3 to MoO_2 occurred at 480 °C for CR and at 385 °C for TPR. At that point, there is also a large positive spike in the heat flow, showing that the oxide reduction is an exothermic process. Thus, similar information on the carburization temperature can be obtained from DTA as well as from TGA. For the CR synthesis profile, the heat flow and the weight loss changed at 710 °C, indicating the formation of the carbide. The negative spike in the heat flow is indicative of an endothermic process. The graph for TPR shows no clear changes in the heat flow at the carburization temperature.

CR of the bare support material (CNF)

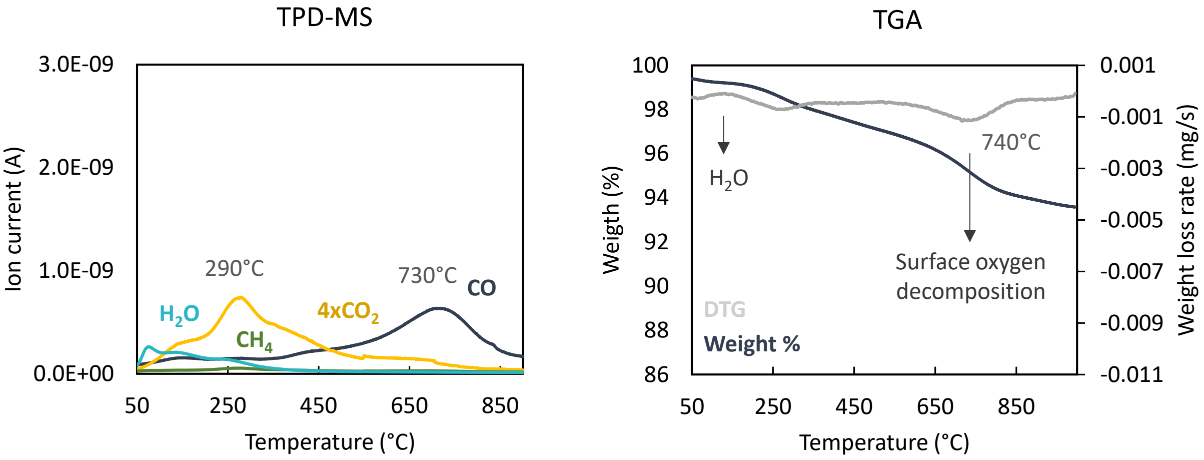

Figure S2. TPD-MS (left) and TGA (right) on CNF heated to 900 ˚C under inert gas.

Figure S2 shows the TPD-MS and TGA results for the CNF support without any precursors during the CR treatment. The quantity of all measured gases (H_2O, CO_2 and CO) was lower than for the samples with metal precursors (vide infra); see Figures S2 and S3. The main CO_2 peak is located at 290 ˚C and the amount of evolved CO_2 decreases with increasing temperature. The main CO peak occurs at 730 ˚C. This release of CO_2 and CO is due to the loss of different types of surface oxygen groups from the CNF.[1] A similar trend is visible in the TGA results. The CNF lost 6 wt% in total, which is significantly less than the weight loss for the samples with metal precursors (~12 wt%, figure) but does show that decomposition of the support itself likely also contributes to the measured mass losses observed during the CR treatment.

Mass loss during CR synthesis

Table S1 shows the assumed reactions during the CR synthesis with calculated theoretical normalized mass loss and measure normalized mass loss (%) at the indicated temperature ranges.

Table S1. Theoretical and measured mass losses during the CR carburization of the monometallic Mo/CNF and W/CNF.

Synthesis step	Calculated Mass loss (%)	Measured Mass loss(%)	Temp. range (˚C)
**Precursor decomposition:**			
$(NH_4)_6Mo_7O_{24}$ *$4H_2O$→ $7MoO_3$ + $7H_2O$ + $6NH_3$	2.7	2.1	150-350
$(NH_4)_6H_2W_{12}O_{40}$ *$4H_2O$→ $12WO_3$ + $8H_2O$ + $6NH_3$	1.6	1.7	150-350
**Oxide reduction:**			
$2MoO_3+C_{CNF}$ → $2MoO_2$ + CO_2	1.8	2.0	350-580
**Carburization:**			
$2MoO_2$+ 5C → Mo_2C + 4CO	4.6	5.4	610-780
$2WO_3$+7C → W_2C + 6CO	6.4	7.1	610-900

TPR for the bare support material (CNF)

In addition to monometallic and bimetallic carbides, the TPR of which we followed by TPD-MS, TGA, and XRD, we also prepared and analyzed a physical mixture of both carbides for the sake of comparison.

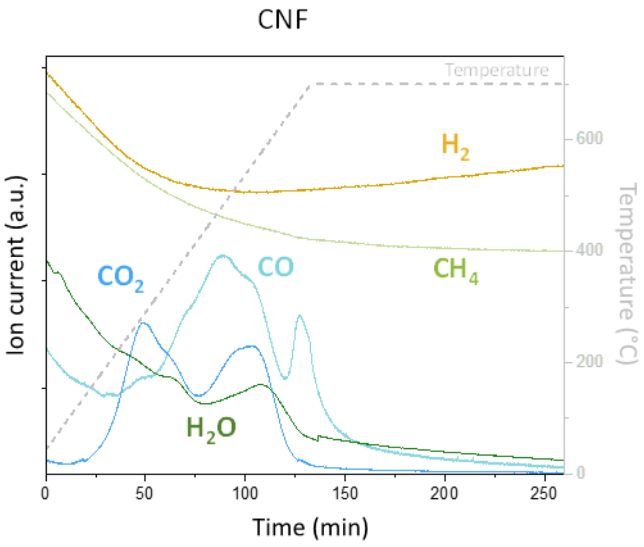

Figure S3. TPR-MS on CNF heated to 700 °C under 20% CH_4/H_2.

Figure S3 shows the TPD-MS of the CNF support without any precursor during the TPR method. This data was obtained to serve as a comparison with the precursor impregnated materials being carburized shown in Figure S6. Gas evolution of CO (m/z=28), H_2O (m/z=18), CH_4 (m/z=16), and CO_2 (m/z=44) was followed. The main CO_2 peak is located at 290 °C and second is located at 500 °C, simultaneously with a water release. The main CO peak occurred at 450 °C; there was further CO release at 690 °C.

Mass loss during TPR synthesis

Table S2 shows the assumed reactions during the TPR synthesis with calculated theoretical normalized mass loss and measure normalized mass loss (%) at the indicated temperature ranges.

Table S2. Theoretical and measured mass losses during the TPR carburization of the monometallic Mo/CNF and W/CNF.

Synthesis step	Calculated Mass loss (%)	Measured Mass loss(%)	Temp. range (°C)
**Pre decomposition:**			
$(NH_4)_6Mo_7O_{24}$ *$4H_2O \rightarrow 7MoO_3 + 7H_2O + 6NH_3$	2.6	2.1	130-350
$(NH_4)_6H_2W_{12}O_{40}$ *$4H_2O \rightarrow 12WO_3 + 8H_2O + 6NH_3$	1.6	1.8	170-350
**Oxide reduction:**			
$MoO_3 + H_2 \rightarrow MoO_2 + H_2O$	1.3	2.2	360-420
$MoO_3 + CH_4 \rightarrow MoO_2 + CO + 2H_2$	1.3	2.2	360-420
$WO_3 + H_2 \rightarrow WO_2 + H_2O$		-	
$WO_2 + 2CH_4 \rightarrow W + 2CO + 4H_2$		-	
Overall: $WO_3 + 2CH_4 \rightarrow W + H_2O + 2CO + 3H_2$	3.6	4.4	450-660
**Carburization:**			
$2MoO_2 + 5CH_4 \rightarrow Mo_2C + 4CO + 10H_2$	2.1	2.0	480-670
$2W + CH_4 \rightarrow W_2C + 2H_2$	-0.4	-0.02	660-700

Element mapping (STEM-EDX)

Figure S4 (CR) and Figure S5 (TPR) show HAADF-STEM and EDX images of the bimetallic carbide system with a Mo:W ratio of 1:3 and 3:1. (The main text includes images of the 1:1 system.) For the monometallic carbides, we obtained TEM images but did not apply any element mapping.

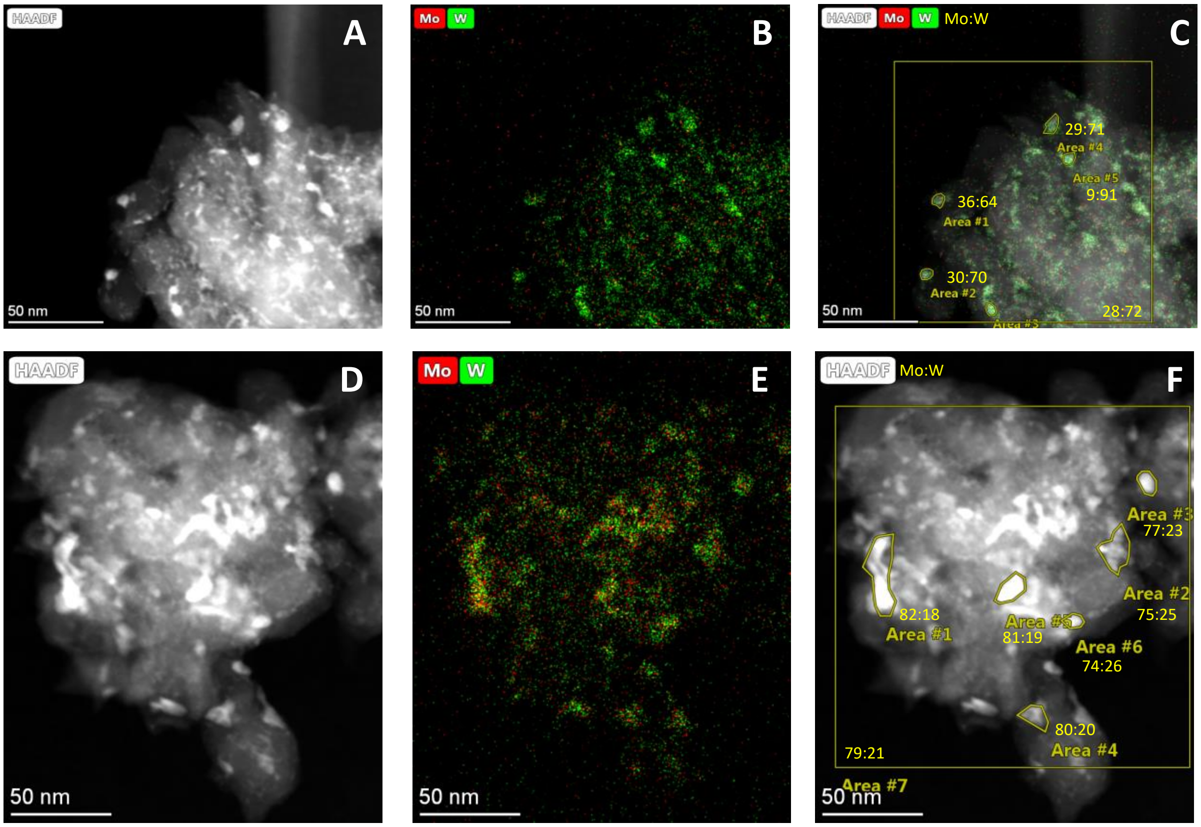

Figure S4. STEM characterizations of the CR-prepared MoWC/CNF samples with a Mo:W ratio of 1:3 (A-C) and 3:1 (D-F)

The 1:3 MoW carbide sample synthesized via the CR method (BET surface area = 133 m^2/g, total pore volume = 0.3 ml/g) showed only nanoparticles that contained both Mo and W. Some particles contained a large proportion of W, e.g. area 5 in Figure S4C, but we observed no particles that contained only Mo or W. The atomic Mo:W ratio (yellow box) of the whole imaged sample was 28:72, which is close to the expected bulk ratio of 1:3. We found similar results for the 3:1 sample synthesized via the CR method (BET surface area = 144 m^2/g, total pore volume = 0.3 ml/g). We detected no particles that contained only Mo or W; all particles were mixed with an atomic Mo:W ratio of approximately 80:20.

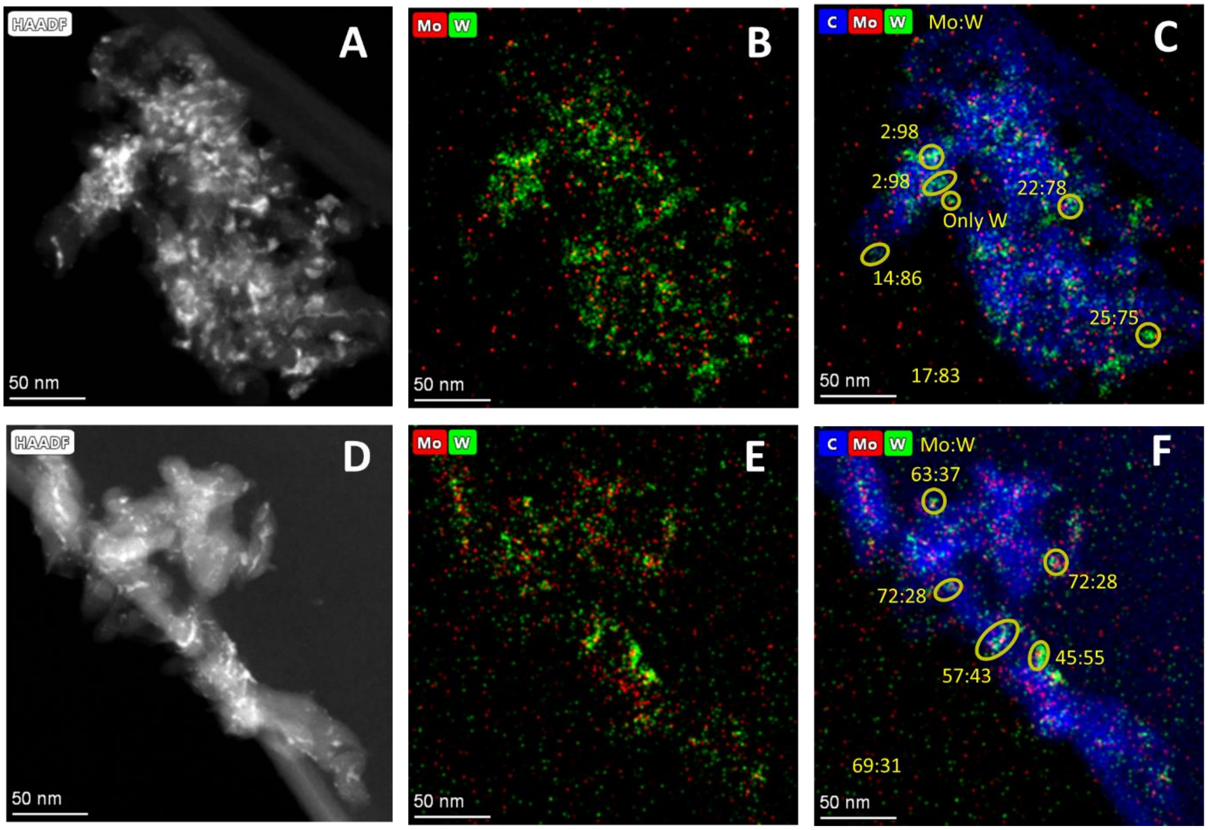

Figure S5. STEM characterizations of the TPR-prepared MoWC/CNF sample with a Mo:W ratio of 1:3 (A-C) and 3:1(D-F).

We made similar observations for samples prepared with the TPR method. For the MoW carbide with a Mo:W ratio of 1:3 (BET surface area = 149 m^2/g, total pore volume = 0.3 ml/g), almost all nanoparticles were bimetallic. However, we found a few small particles that only contained W. The Mo:W ratio over the whole image was 17:83, which again is in agreement with the expected bulk ratio. Also, the 3:1 bimetallic carbide (BET surface area = 141 m^2/g, total pore volume = 0.3 ml/g) showed only nanoparticles containing both metals; the bulk ratio of the whole image was 60:31.

In summary, the bimetallic nanoparticles synthesized via both the CR and the TPR method consisted of a mixed metallic phase. These nanoparticles did not have a homogenous distribution, but overall had an atomic ratio close to the expected bulk ratio.

N_2-physisoption

Table S3.Comparison micropore and mesopore area obtained from N_2-physisoption.

		**Micropore area (m^2/g)**	**Mesopore area (m^2/g)**
**CR**	MoWC/CNF (1:1)	12	0.26
	MoWC/CNF (1:3)	18	0.30
	MoWC/CNF (3:1)	24	0.31
**TPR**	MoWC/CNF (1:1)	20	0.31
	MoWC/CNF (1:3)	31	0.33
	MoWC/CNF (3:1)	29	0.32

Since the different synthesis temperatures (900 °C for CR and 700 °C for TPR) might have an effect on the porosity of the CNF support, which in turn could results in different mass transfer properties, we investigated the micropore and mesopore area with N_2-physisoption (see Table S3). We found that both synthesis methods lead to a comparable micropore and mesopore areas.

Evaluation of the XRD carbide reflections

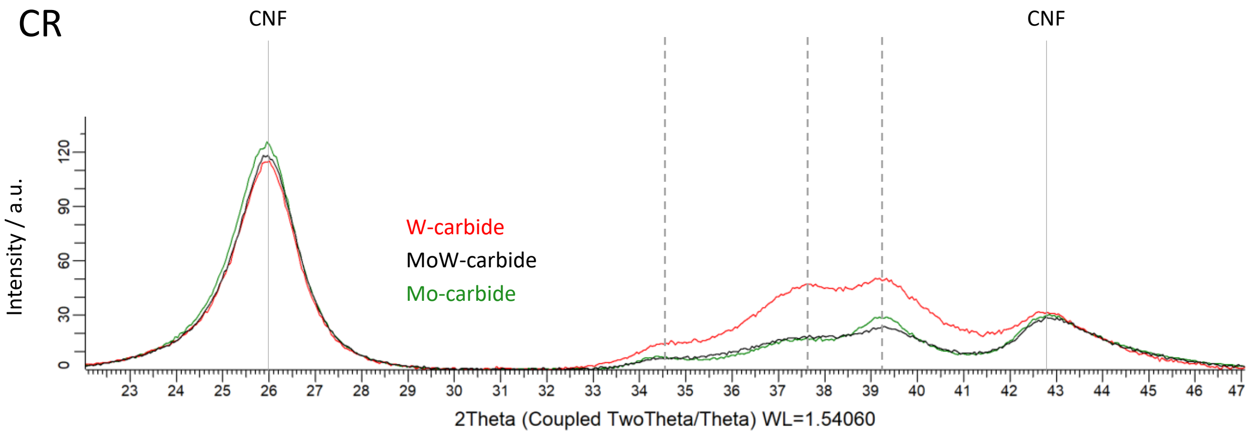

Peak position	CNF position	Carbide	Carbide	Carbide
Mo_2C/CNF	25.991	34.434	37.747	39.219
$(Mo,W)_xC$/CNF (3:1)	25.990	34.624	37.791	39.381
$(Mo,W)_xC$/CNF (1:1)	25.992	34.550	37.590	39.322
$(Mo,W)_xC$/CNF (1:3)	25.995	34.653	37.747	39.276
W_2C/CNF	25.992	34.585	37.808	39.179

Figure S6. XRD patterns of CR-carburized carbides in the 2ϑ range of 22 to 47°.

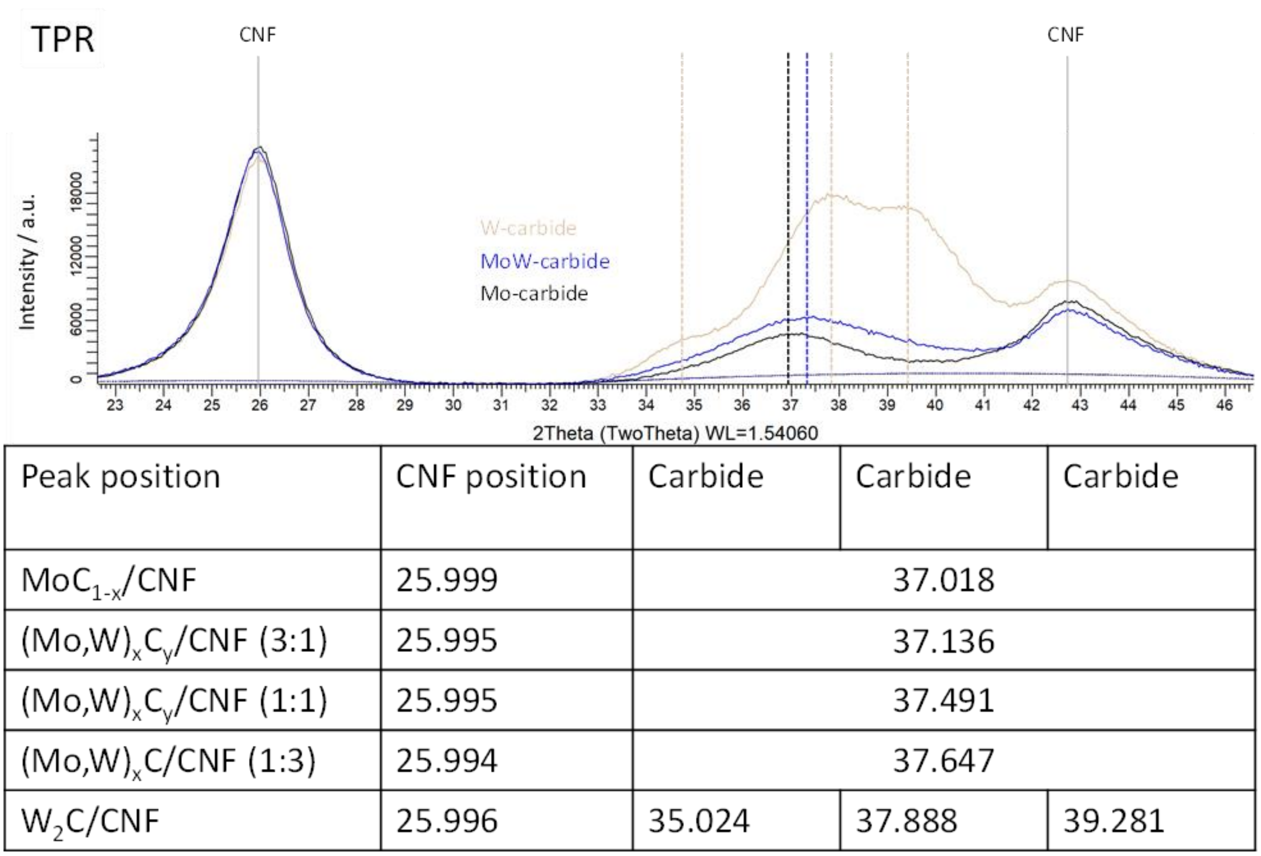

Peak position	CNF position	Carbide	Carbide	Carbide
MoC_{1-x}/CNF	25.999	37.018		
$(Mo,W)_xC_y$/CNF (3:1)	25.995	37.136		
$(Mo,W)_xC_y$/CNF (1:1)	25.995	37.491		
$(Mo,W)_xC$/CNF (1:3)	25.994	37.647		
W_2C/CNF	25.996	35.024	37.888	39.281

Figure S7. XRD patterns of TPR-carburized carbides in the 2ϑ range of 22 to 47°.

Figure S6 and S7 show the XRD patterns of the carbide catalysts. The peaks were corrected for sample height displacement by aligning all the (002) CNF peaks to 25.99°. The experimentally obtained XRD data was compared with reference patterns to determine the (main) carbide phase. The references which had the best match in terms of position and intensity are listed below:

- CR-carburized Mo_2C pattern (PDF 79-0744) 34.359°, 37.975° and 39.413°
- CR-carburized W_2C pattern (PDF 89-2371) 34.459°, 38.034° and 39.519°
- TPR-carburized MoC_{x-1} pattern (PDF 65-0280) 36.388°
- TPR-carburized W_2C pattern (PDF 79-0743) 34.536°, 38.067° and 39.592°

It has been suggested that Vegard's law can be used to prove the formation of a mixed-metal carbide phase. [2] Figure 12 reveals that the positions of the carbide peaks are indeed between those of the monometallic carbides. However, the expected systematic shift in peak position with increasing Mo is absent and there is no clear relationship between the peak position and the Mo:W ratio. This could be because the difference between

β-Mo_2C and β-W_2C is intrinsically small (0.1 2θ) and since the mixed-metal nanoparticles yield very broad diffraction peaks, this probably results in a relatively large uncertainty for the exact peak position.

Also for the TPR method, we are hesitant to claim proof of a mixed carbide phase based on the XRD data; see Figure S13. The W carbide forms a hexagonal crystal structure and the Mo carbide a cubic structure. The bimetallic carbides show reflections best corresponding to the cubic structure. With increasing W content, a shift towards a greater diffraction angle is observed for these samples, which could be due to the formation of a mixed phase. However, we cannot exclude that the sample contains an increasing fraction of the hexagonal structure the reflection of which could be part of the broad reflection peak at 37°. If so, it would also cause this broad reflection to appear to shift to a greater angle.

Hydrogen mass transfer limitations

Since the reactions were conducted in a batch stirred reactor, possible mass transfer limitations should be considered. Figure S8 shows the HDO reaction of Mo carbide catalysts at 300 °C (left) and 350 °C (right) at different stirring rates. As can be seen from the 300 °C experiment, the stearic acid conversion is lower for lower stirring rates (400 rpm), indicating that mass transfer rate of hydrogen is rate limiting. Upon increasing the stirring rate to 800 and 1200 rpm the stearic acid conversion does increase. However, when the stirring is further increased to 1600 rpm, the conversion seems to decrease. A similar trend was observed for the HDO at 350 °C. Thus it was concluded that 800 rpm suffices for the reaction.

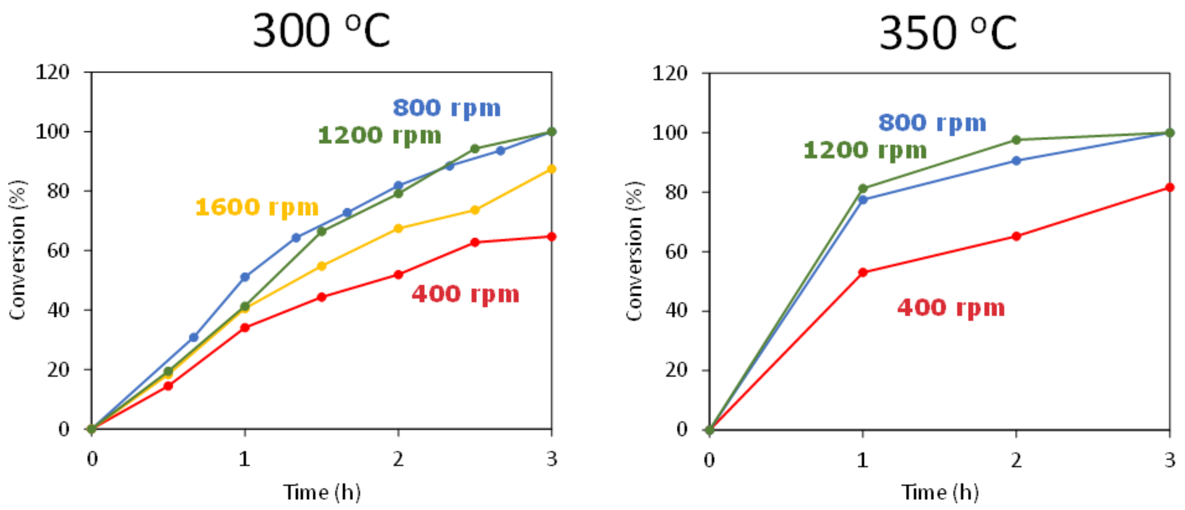

Figure S8. Effect of stirring rate on the stearic acid conversion at 300 °C and 350 °C. (250 mg catalyst with metal loading of 0.9 mol/$g_{catalyst}$, 2 g stearic acid, 50 ml solvent, 30 bar H_2)

Reproducibility

Two separate reaction runs were performed using newly synthesized monometallic and the MoW (1:1) carbide catalyst batches in order to test the experimental reproducibility. Figure S9 the average stearic acid conversion with the standard deviation of the two runs is shown. Based on these results we conclude that the synthesis and experiments were reproducible.

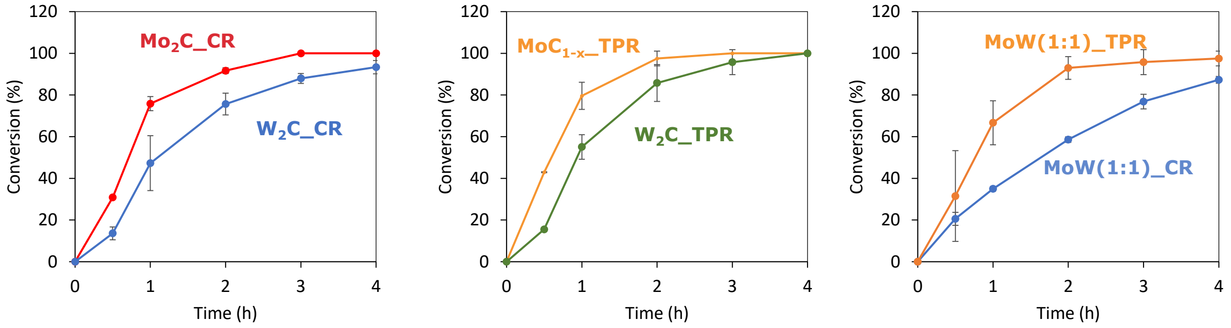

Figure S9. Average activity of two separate HDO runs (250 mg catalyst with metal loading of 0.9 mmol/$g_{catalyst}$, 2 g stearic acid, 350 °C, 50 ml solvent, 30 bar H_2)

References

1. Rodrigues, E. G.; Pereira, M. F. R.; Chen, X. W.; Delgado, J. J.; Orfao, J. J. M., Influence of activated carbon surface chemistry on the activity of Au/AC catalysts in glycerol oxidation. *Journal of Catalysis* **2011,** *281* (1), 119-127.
2. Fu, Q.; Peng, B. X.; Masa, J.; Chen, Y. T.; Xia, W.; Schuhmann, W.; Muhler, M., Synergistic Effect of Molybdenum and Tungsten in Highly Mixed Carbide Nanoparticles as Effective Catalysts in the Hydrogen Evolution Reaction under Alkaline and Acidic Conditions. *Chemelectrochem* **2020,** *7* (4), 983-988.
